# Deciding While Acting—Mid-Movement Decisions Are More Strongly Affected by Action Probability than Reward Amount

**DOI:** 10.1523/ENEURO.0240-22.2023

**Published:** 2023-04-17

**Authors:** Philipp Ulbrich, Alexander Gail

**Affiliations:** 1 Cognitive Neuroscience Laboratory, German Primate Center—Leibniz Institute for Primate Research, 37077 Göttingen, Germany; 2 Faculty of Biology and Psychology, Georg-August University, 37073 Göttingen, Germany; 3 Bernstein Center for Computational Neuroscience, Georg-August University, 37073 Göttingen, Germany; 4 Primate Cognition, Leibniz ScienceCampus, 37077 Göttingen, Germany

**Keywords:** action, deciding while acting, decision-making, psychophysics, reaching

## Abstract

When deciding while acting, such as sequentially selecting targets during naturalistic foraging, movement trajectories reveal the dynamics of the unfolding decision process. Ongoing and planned actions may impact decisions in these situations in addition to expected reward outcomes. Here, we test how strongly humans weigh and how fast they integrate individual constituents of expected value, namely the prior probability (PROB) of an action and the prior expected reward amount (AMNT) associated with an action, when deciding based on the combination of both together during an ongoing movement. Unlike other decision-making studies, we focus on PROB and AMNT priors, and not final evidence, in that correct actions were either instructed or could be chosen freely. This means, there was no decision-making under risk. We show that both priors gradually influence movement trajectories already before mid-movement instructions of the correct target and bias free-choice behavior. These effects were consistently stronger for PROB compared with AMNT priors. Participants biased their movements toward a high-PROB target, committed to it faster when instructed or freely chosen, and chose it more frequently even when it was associated with a lower AMNT prior than the alternative option. Despite these differences in effect magnitude, the time course of the effect of both priors on movement direction was highly similar. We conclude that prior action probability, and hence the associated possibility to plan actions accordingly, has higher behavioral relevance than prior action value for decisions that are expressed by adjusting already ongoing movements.

## Significance Statement

Natural behavior, like foraging or hunting prey, requires animals and humans to select their next action during ongoing movements, thereby updating movements as the decision process unfolds. Here, we study the magnitude and time course with which prior action probability and prior expectancy of reward amount influence the selection between two competing movements in humans. By simultaneously but independently manipulating both priors in individual decisions, and by avoiding confounds of reward probability, we show that both priors affect the decision process with different magnitude yet comparable time courses. Our results emphasize the prioritized relevance of action probabilities over action values on mid-movement decisions.

## Introduction

Everyday decisions often are not abstract (e.g., which university courses to select) but are immediately linked to action alternatives (e.g., whether to bypass an oncoming person on the sidewalk left or right). For such embodied decisions (Pezzulo and Cisek, 2016; Gail, 2022), it has been shown that action selection, and action preparation and control are parallel, interconnected processes that are at least partially governed by overlapping mechanisms (Nashed et al., 2014; Morel et al., 2017; Carroll et al., 2019) and shared neural structures (Cisek and Kalaska, 2005; Klaes et al., 2011; Pastor-Bernier and Cisek, 2011; Suriya-Arunroj and Gail, 2019). They allow us to make and revise decisions online (i.e., during ongoing movements; Resulaj et al., 2009; Friedman et al., 2013; Atiya et al., 2020; Michalski et al., 2020; for review, see Cisek and Kalaska, 2010; Gallivan et al., 2018; Wispinski et al., 2020; Kim et al., 2021). The desirability of an option, and hence the likelihood of it being selected, can be described via its expected value (EV; i.e., the product of its rewards and the probability of obtaining these outcomes; Trommershäuser et al., 2006, 2008; Stillman et al., 2020). Yet, the likelihood of a choice determines the plannability of the associated action, making it difficult to assess the respective contributions of reward amount (AMNT) and probability (PROB) independently in embodied decision-making (Suriya-Arunroj and Gail, 2015). Here, we disentangle reward amount (preferability) from action probability (plannability) and ask how probability and reward priors independently affect movement kinematics and are dynamically integrated during online decisions.

Previous studies demonstrated how differences in prior reward AMNT or prior PROB between optional movements lead to preparatory motor activity and its behavioral correlates, such as reduction in movement initiation time and early biases in movement direction, favoring the higher rewarded or more probable action over its alternatives (Platt and Glimcher, 1999; Chapman et al., 2010; Pastor-Bernier and Cisek, 2011; Suriya-Arunroj and Gail, 2019; Marti-Marca et al., 2020). By “priors,” we refer to information that is already available at the start of the decision process (Gold and Shadlen, 2007; i.e., before movement initiation) but does not yet provide evidence for the final reward contingencies. Priors might be induced by short-term visual cueing (Leis et al., 2005; Chapman et al., 2010; Suriya-Arunroj and Gail, 2015) or be highly internalized by learning over many hundred trials (Körding and Wolpert, 2004; Seydell et al., 2008). However, in these previous studies, only one of the two prior types was applied in each, preventing relative weighing of the impact on online decisions of either prior. Here, we use an online spatial selection paradigm and apply both priors simultaneously to study how strongly and at which time course (Scherbaum et al., 2010; Dotan et al., 2019; Scherbaum and Dshemuchadse, 2020) prior probability and reward expectancy affect movement and choice behavior within the same decision.

Applying both, PROB and AMNT priors within the same decision has potentially confounding effects on the EV because the AMNT prior typically affects both reward magnitude and probability. In free-choice paradigms, participants choose higher valued options with higher probability (Marti-Marca et al., 2020). Here, we made the probability of obtaining a reward independent from the AMNT prior by combining rewarded instructed trials with value-neutral (i.e., unrewarded) free-choice trials, similar to those in a previous study by Suriya-Arunroj and Gail (2015). There, the authors showed that PROB priors drive action planning and subsequent choice to a much larger degree than AMNT priors, postulating that PROB priors influence the decision process earlier than AMNT priors as the latter do not suffice to bias action planning between choice options. In contrast to the current study, Suriya-Arunroj and Gail (2015) applied either prior in separate experiments and participants were required to withhold their movement until after a target was instructed or chosen, thereby preventing the authors from directly measuring both the postulated temporal differences between the effects of each prior as well as the integration of both priors within the same decision.

We hypothesize, first, that the PROB prior biases both movements and choices in favor of higher PROB targets more strongly than the AMNT prior does in favor of higher AMNT targets (both compared with lower PROB/AMNT alternatives), and that the effect of the PROB prior on the movements emerges earlier than the effect of the AMNT prior. We further ask whether such dominance of PROB priors compared with AMNT priors persists even if the higher PROB target is associated with a lower EV (by means of combining it with a low-AMNT prior) than the lower PROB alternative, as this would argue for a higher behavioral relevance of target plannability over target preferability in decisions during ongoing movements.

## Materials and Methods

### Participants

Twenty participants (14 female; mean age, 24.4 years; age range, 19–32 years; all were right handed; all had normal or corrected-to-normal vision), who were recruited via the internal jobs board of the university, took part in this study. Six participants had participated in similar experiments before, but all participants were naive with respect to the purpose of the current study. Each participant completed three sessions (one training session, two test sessions) on 3 separate days and was paid a fixed remuneration plus a performance-dependent bonus (calculated from tokens they gained throughout the experiment; see below). An additional eight participants did not complete the study, of which seven were not able to complete the training and one opted out after the training. All participants gave their written informed consent before participation. Before the training session, participants received written instructions (including step-by-step task illustrations) specifically tailored to the trial design. Participants were additionally given the opportunity to review the appropriate instructions at the start of the main experiment sessions and were encouraged to ask the experimenter questions if things remained unclear. The experiment was performed in accordance with institutional guidelines for experiments with humans, adhered to the principles of the Declaration of Helsinki, and were approved by the ethics committee of the Georg Elias Mueller Institute for Psychology at the University of Göttingen.

### Apparatus

**Figure 1.**
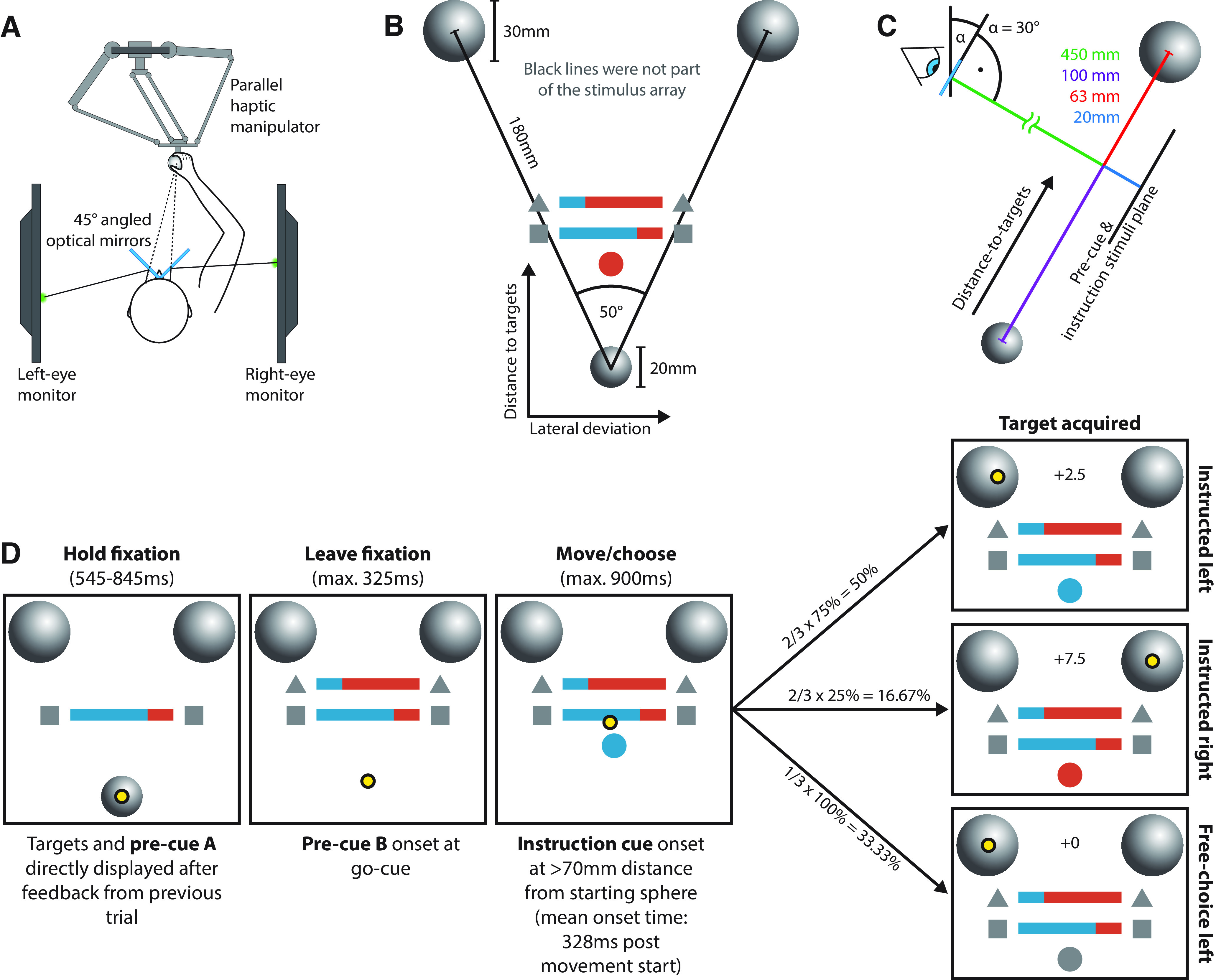
Apparatus, stimuli, behavioral paradigm. ***A***, Subjects performed reaching movements using a parallel haptic manipulator and perceived all visual stimuli as projected into the manipulator workspace via a stereoscopic 3D-AR setup. ***B***, Visual stimuli (drawn to scale). The position of the starting (bottom) and target (top) spheres defined a stimulus plane, which we describe using the terms “lateral deviation” (corresponds to *x*-axis) and “distance to targets” (corresponds to *y*-axis). The PROB/AMNT pre-cues (colored bars) and the instruction cue (colored disk) were set on a parallel stimulus plane 20 mm behind the previously described plane. ***C***, Viewing angle of the stimulus planes. The monitors and mirrors of the AR setup were angled by 30° relative to the vertical to lower the visual stimuli into the manipulator workspace. ***D***, Example trial structure. Participants performed reaching movements toward two potential targets and were either instructed mid-movement to reach toward a specific target (instructed trial, two-thirds of all trials) or were allowed to freely choose between the targets (free-choice trial, one-third of all trials). Participants initiated a trial by moving the yellow cursor into the starting sphere and keeping it there for the duration of the then initiated Hold fixation period. Following this period, an auditory go-cue signaled the participants to quickly initiate their movement toward the array of targets (Leave fixation). Starting before the Hold fixation and from the start of the Leave fixation periods, respectively, two pre-cues were displayed. The PROB precue (here: precue A) informed participants about the relative probability with which either target was instructed in case of an instructed trial (here: left/right = 75%/25%). The AMNT precue (here: precue B) informed participants about the reward amount that was obtained on successfully following the instruction (here: left/right = 2.5/7.5 tokens). Starting the movement during the Leave fixation period initiates the Move/choose period. Throughout the study, movement times are defined relative to the start of the Move/choose period. During the Move/choose period, after moving away from the starting sphere by >70 mm, the instruction cue either instructed the participants to reach to either the left or right target (here: left) or to freely choose between the targets. Upon reaching the instructed target/freely chosen target, the participants received feedback with regard to the number of reward tokens they obtained (Target acquired). As free choices were value neutral, reaching a freely chosen target always yielded zero reward tokens regardless of the AMNT precue. In the actual experiment, the stimuli were presented on a black background, and the stimuli indicating the value cue type and the free-choice cue were white. See Extended Data Table 1-1 for all possible PROB and AMNT levels and their frequencies of occurrence per experimental session.

The participants performed reaching movements using a parallel-type haptic manipulator (model delta.3, Force Dimension) inside a 3D augmented reality (3D-AR) environment (Fig. 1*A*). The manipulator was connected to a computer running custom software (C++, OpenGL), which was responsible for task control, including visual stimulus generation, hand position recording (manipulator handle position sampled at 2 kHz), and task event recording (digital input/output). The 3D-AR environment consisted of two computer monitors [screen size, 590 × 338 mm; refresh rate, 60 Hz; viewing distance, 47 mm; model XL2720T, BENQ (with DualHead2Go Display Port Splitter, Matrox)] that were viewed through a pair of semitransparent mirrors, tilted 45° relative to the screens. Subjects only viewed one screen per eye, which allowed for the creation of stereoscopic 3D images perceived as directly projected into the manipulator workspace. This means that all movement-related stimuli such as movement starting points and targets (Fig. 1*B*) were directly presented at their supposed physical location. The position of the manipulator handle was represented in the 3D-AR environment as a yellow sphere cursor (*d* = 6 mm) at its actual physical location. Display and manipulator latencies were compensated by a forward prediction using a Kalman filter with position, speed, and acceleration as state variables to synchronize the movement of the handle and the cursor. The haptic manipulator was mounted approximately at chest height to allow for comfortable operation. Consequently, the monitors and the mirror were additionally tilted by 30° to lower the 3D representation into the manipulator workspace (Fig. 1*C*, angle α).

10.1523/ENEURO.0240-22.2023.tab1-1 Table 1-1 Trial numbers per session and block for each PROB/AMNT combination. ^a^Left, Right instruction probability; ^b^left, right reward amount; ^c^number of required hits for left instructed/right instructed/free-choice. †Each row equals one block type. Each block type (PROB condition) is repeated four times. ‡Each column equals one block type. Each block type (AMNT condition) is repeated two times. Download Table 1-1, DOCX file.

### Behavioral paradigm

Participants performed a go-before-you-know reaching task in which they moved a cursor from a starting sphere at the bottom of a stimulus plane toward one of two reach targets placed at the top of the stimulus plane (Fig. 1*B–D*). In two-thirds of all trials, participants were instructed which target to acquire (the “instructed trial”). In the remaining randomly interspersed third of all trials, participants freely chose between the two targets (the “free-choice trial”). Importantly, the instruction cue, which indicated either which target must be selected or whether participants were to freely choose, appeared approximately halfway into the movement. This allowed participants to commit to a target only after movement initiation and required them to adjust their movement direction online.

The targets were additionally characterized by two prior pre-cues (PROB pre-cue and AMNT pre-cue), which were both presented at different time points before movement onset (see subsecion Trial structure). The PROB pre-cue informed the participants about which target was more likely to be instructed, and the AMNT pre-cue informed the participants about the number of reward tokens associated with either target in case it was instructed to be acquired. If the trial turned out to be free-choice, both targets were unrewarded (i.e., the free-choice was value neutral). For improved readability, the AMNT conditions are always referred to by their associated pre-cue values, regardless of whether the target was instructed or freely chosen. For instance, “AMNT = 9 free-choices” refers to trials in which participants chose the target that would have been rewarded with 9 tokens if instructed, although in free-choice trials the reward was zero. Both pre-cues consisted of a horizontal bar that was split vertically in proportion to the respective pre-cue values, and each side was colored differently (Fig. 1*B*,*D*). For instance, if the AMNT pre-cue indicated a 9:1 (left/right) reward distribution, the left portion made up 90% of the total width of bar. The color–side mapping (i.e., whether left was blue and right was orange, or vice versa) was randomized between trials, but kept constant across pre-cues (i.e., if the left portion of the AMNT pre-cue was blue, so was the left portion of the PROB pre-cue). To enable participants to tell apart the pre-cues, the PROB pre-cue was flanked by two squares and the AMNT pre-cue was flanked by two triangles (Fig. 1*B*,*D*).

#### Trial structure

Each participant completed a training session (see below) and two main experiment sessions (session 1 and session 2). In session 1, 50% of the participants each received either the PROB pre-cue or the AMNT pre-cue already at the start of each trial (Fig. 1*D*, pre-cue A, horizontal bar). Upon trial start, participants moved the cursor into the fixation sphere and kept it there for a uniformly randomized hold period of 545–845 ms (Fig. 1*D*, hold fixation). Following this hold period, an auditory go-cue prompted the participants to initiate their movement as quickly as possible (Fig. 1*D*, leave fixation). Precue B (AMNT if pre-cue A was PROB and vice versa) was displayed simultaneously with the go-cue. In session 2, the order of the PROB and AMNT pre-cues was reversed (e.g., session 1 pre-cue A = PROB and pre-cue B = AMNT resulted in session 2 pre-cue A = AMNT and pre-cue B = PROB).

The Move/choose stage (Fig. 1*D*, left) was initiated on movement start. The task-controlling software was programmed to register a movement start either at the instance the cursor left the starting sphere or when the cursor was accelerated to at least 0.02 m/s still within the starting sphere, whichever happened first. The latter criterion was introduced to obtain a more accurate estimate of the movement start (e.g., in situations where the cursor was placed close to the lower edge of the starting sphere and thus was already being moved up to 20 mm before leaving the starting sphere). Once the participants moved away, 70 mm from the starting sphere in any direction, the display of the instruction cue was triggered, which became visible on average 45 ms later (translating into mean ± SD = 283 ± 35 ms post-movement initiation; Fig. 1*D*, move/choose, colored disk below the pre-cues). In instructed trials, the instruction cue matched the color of either the left or the right side of the pre-cues, and participants were required to move toward the corresponding left/right target. In free-choice trials, the instruction cue was white, and participants could freely choose between the targets. Once the participants acquired the appropriate target, onscreen feedback informed them about how many tokens they had acquired (Fig. 1*D*, target acquired). The tokens obtained throughout the experiment were converted into a performance-dependent bonus remuneration (see subsection Participant remuneration). Participants received detailed error feedback if they failed a trial (initiating the movement outside of the “Leave fixation” window: “too early”/”too late”; stopping the movement outside the target before the movement time window had expired: “aborted”; not reaching the target in time: “too slow”; selecting the wrong target in instructed trials: “wrong target”).

#### Task conditions

The PROB pre-cue was manipulated on three levels (left target vs right target instruction probability = 0.25:0.75, 0.5:0.5, 0.75:0.25). These instruction probabilities were conditioned on the trial being instructed in the first place. Since two-thirds of all trials were instructed, a 0.75:0.25 PROB pre-cue, for example, indicated that the left and right targets had a 2/3 × 0.75 = 50% and 2/3 × 0.25 = 16.67% absolute instruction probability, respectively. The AMNT pre-cue was manipulated on five levels (left target tokens vs right target tokens = 1:9, 2.5:7.5, 5:5, 7.5:2.5, 9:1). All possible combinations of PROB × AMNT × location (left/right) of the high-PROB option × location of the high-AMNT option were presented to the participants. For all analyses, we pooled the data of each PROB ratio and each AMNT ratio across target locations (i.e., the 0.25:0.75 and 0.75:0.25 conditions were pooled, and identically the 1:9 and 9:1 conditions, 2.5:7.5 and 7.5:2.5 conditions, and so forth).

Precue A was always blocked [i.e., its PROB (or AMNT) ratio was kept identical for a set number of successful trials]. Precue B was randomized on a per-trial basis. Each main experiment session consisted of 720 successful trials. The number of trials per condition (which differed between conditions as it depended on the PROB pre-cue) was identical across sessions, but the number of trials per block differed depending on which pre-cue was blocked. In the pre-cue A/B = PROB/AMNT session, participants completed 12 blocks of 60 successful trials each. In the pre-cue A/B = AMNT/PROB session, participants completed 10 blocks of 72 successful trials each (Extended Data Table 1-1, complete description of the per-condition number of trials per session, per pre-cue A = PROB block, and per pre-cue A = AMNT block).

Our task-controlling software randomized the block order within each session and the trial order within each block. Unsuccessful trials were reinserted into the randomizer and repeated at a random location within the same block. Additionally, we randomized, per-trial, the color-side mapping (blue = left and orange = right vs vice versa), and the pre-cue location (pre-cue A on top of pre-cue B vs vice versa).

#### Dissociation of PROB and the influence of AMNT on expected value

By means of either instructing the target or allowing value-neutral free choices, we dissociated the influence of the PROB and AMNT priors on the EVs of reach targets (expected value = reward probability × reward amount) and subsequently their influence on the participants’ movement and choice behavior. Note that by EV, we refer to the preliminary EV at the time point of the pre-cues and independent of the instruction. Receiving a reward on acquiring an appropriate target was deterministic (i.e., reaching an instructed target always resulted in the pre-cued reward), and reaching a target in free choice always resulted in no reward. We designed our experiment such that, if only successful trials were considered, the preliminary EV was only determined by the product of PROB × AMNT, and the probability of obtaining a reward was only determined by the PROB pre-cue. How value-neutral free choices dissociate the influence of PROB and AMNT on this preliminary EV is illustrated in the following numerical examples comparing the same scenario either with value-neutral (unrewarded) or reward-associated choice trials.

##### Scenario A, reward-associated, not used in experiment

We consider how the preliminary EVs and preliminary reward probabilities (at the time point of the pre-cues) would look like if the free choices were rewarded according to the AMNT pre-cue, as were the instructed trials. The preliminary EV for the left target (and right target correspondingly) would be as follows:

$${\text{EV}}_{\text{left}}=p\left(\text{instructed trial}\right)\times {\text{PROB}}_{\text{left}}\times {\text{AMNT}}_{\text{left}}+p(\text{free choice trial})\times p(\text{left chosen})\times {\text{AMNT}}_{\text{left}}.$$

For a reward-maximizing decider who always chooses the higher rewarded target in free-choice trials, the preliminary EV for trials with PROB_left_/PROB_right_ = 0.75/0.25 and AMNT_left_/AMNT_right_ = 2.5/7.5 would be the following:

$${\text{EV}}_{\text{left}}=\frac{2}{3}\times 0.75\times 2.5+\frac{1}{3}\times 0\times 2.5=1.25,$$

$${\text{EV}}_{\text{right}}=\frac{2}{3}\times 0.25\times 7.5+\frac{1}{3}\times 1\times 7.5=3.75.$$

Here, the less rewarded left target is never chosen in free-choice trials. The preliminary probability of obtaining a reward for choosing either target in this example would be the following:

$$p\left({\text{reward}}_{\text{left}}\right)=\frac{2}{3}\times 0.75+\frac{1}{3}\times 0=0.5,$$

$$p\left({\text{reward}}_{\text{right}}\right)=\frac{2}{3}\times 0.25+\frac{1}{3}\times 1=0.5.$$

##### Scenario B, value-neutral, used in this study

We consider the same example including a decider who always chooses the target associated with the higher AMNT prior while free choices are always unrewarded. Accordingly, the free-choice portions of the EV and reward probability terms are omitted, as they always equal zero. The preliminary EVs and reward probabilities are as follows:

$${\text{EV}}_{\text{left}}=\frac{2}{3}\times 0.75\times 2.5=1.25,$$

$${\text{EV}}_{\text{right}}=\frac{2}{3}\times 0.25\times 7.5=1.25,$$

$$p\left({\text{reward}}_{\text{left}}\right)=\frac{2}{3}\times 0.75=0.5,$$

$$p\left({\text{reward}}_{\text{right}}\right)=\frac{2}{3}\times 0.25=0.1\bar{6}.$$

These scenarios illustrate how only value-neutral free choices ensure that the preliminary EV is solely governed by the product of PROB and AMNT, and the probability of reward is solely governed by the PROB pre-cue. Considering the $\frac{\text{left target}}{\text{right target}}$ ratios of any of these quantities, we see that only in scenario B

$$\frac{{\text{EV}}_{\text{left}}}{{\text{EV}}_{\text{right}}}=\frac{1.25}{1.25}=1$$

equals

$$\frac{{\text{PROB}}_{\text{left}}\times {\text{AMNT}}_{\text{left}}}{{\text{PROB}}_{\text{right}}\times {\text{AMNT}}_{\text{right}}}=\frac{0.75\times 2.5}{0.25\times 7.5}=1$$

and

$$\frac{p({\text{reward}}_{\text{left}})}{p({\text{reward}}_{\text{right}})}=\frac{0.5}{0.1\bar{6}}=3$$

equals

$$\frac{{\text{PROB}}_{\text{left}}}{{\text{PROB}}_{\text{right}}}=\frac{0.75}{0.25}=3.$$

In scenario A, instead, the preliminary EV and the preliminary reward probability are both skewed toward the higher rewarded right target. In other words, rewarding the free choices according to the AMNT pre-cue would cause the reward amount to affect the reward probability by means of participants choosing the target associated with the higher AMNT more frequently than its alternative in free-choice trials. The influence of choice probability on reward probability in scenario A generalizes to all other cases where the decider chooses either target with a probability that deviates from the instruction probability as determined by the PROB pre-cue. Not rewarding the free-choice trials avoids this confound and dissociates the influence of PROB and AMNT on the preliminary EV.

#### Participant remuneration

Participants completed a training session (∼90–120 min), which was remunerated with 8€/h and two main sessions (∼70 min each) for which participants received 18€ in total. Additionally, participants received a performance-dependent bonus, calculated from the tokens earned in the main sessions. Since participants had to complete a fixed number of successful trials per conditions and free-choice trials were unrewarded, participants always earned 2400 tokens per session. To increase the behavioral relevance of the AMNT manipulation, we therefore additionally considered the number of temporarily missed tokens when calculating the per-session bonus payment, as follows:

$$\mathrm{Bonus}\left(€\mathrm{cents}\right)=0.2\times \frac{\mathrm{total}\;\mathrm{tokens}\;\mathrm{earned}}{\mathrm{total}\;\mathrm{tokens}\;\mathrm{earned}+\mathrm{total}\;\mathrm{tokens}\;\mathrm{missed}}.$$

Missed tokens were defined as tokens participants (temporarily) missed out on because they failed a trial (e.g., if an experiment had consisted of two successful trials rewarded with 5 tokens each and a participant needed three attempts, the resulting bonus would be $0.2\times \frac{10}{10+5}=1.3€$ cents instead of 2€ cents at a 100% hit rate). Here, trials only failed at least 100 ms after the task controller commands to display the instruction cue were considered (i.e., trials where the number of tokens on successful completion was already known to the participant).

### Data analysis

#### Hand tracjectory preprocessing

All data analyses and visualization were conducted using MATLAB 2015b and the *gramm* plotting toolbox for MATLAB (Morel, 2018). For all offline analyses, movement start was defined using the same criteria as applied online during the experiment by the task-controlling software (see above). The end of the movement was defined as the first data point inside the target sphere. As position data we used the movement trajectories projected onto the task-relevant 2D plane defined by the starting and target spheres (Fig. 1*B*,*C*; X, lateral deviation; Y, distance from start) We computed movement speed from the physical 3D velocity. We obtained the movement velocity by differentiating the raw position data. We filtered both the position and velocity data to remove high-frequency noise (fourth-order Butterworth low-pass filter with 12 Hz cutoff, and forward and reverse filtering using the MATLAB *filtfilt* function; per-trial data window of analysis, 1200 ms preceding movement onset until 1200 ms after the end of the movement). We resampled the filtered data to up to 301 data points from 0 (movement start) to up to 900 ms (depending on the actual movement duration) with a bin size of 3 ms to align the data for the time-continuous multiple regression analysis (see below).

#### Data exclusion and pooling

We included all successful trials in our analyses. To test whether trials that directly followed failed trials affect our results, we reran all core analyses on a subset of the data that included only successful trials that did not follow-up failed trials (cross-participant average, 81%). On average, participants failed 22% of all trials, of which on average 75% were failed after movement initiation (stopping mid-movement, not reaching the target in time, reaching the wrong target). Note that failed trials were repeated at randomly chosen later time points and thus the number of successful trials per participant and condition remained constant (see above; Extended Data Table 1-1). The results (not reported) were highly similar to the results performed on the full dataset and led to the same conclusions. Therefore, we included all data in our analyses.

We were interested in the effects of PROB and AMNT independent of their temporal order of cueing. The sequential cueing of PROB and AMNT, and the block-wise manipulation of whichever pre-cue was presented first, was only implemented to make the behavioral task easier for the participants (i.e., to facilitate the processing of both cues by temporally separating their onset and making one of the pre-cues predictable). Consequently, we pooled the data across sessions (i.e., cueing order) for all analyses unless noted otherwise.

#### Time-continuous analysis of PROB and the influence of AMNT on movement trajectories

We asked whether the PROB prior had a larger influence than the AMNT prior in biasing the movement trajectories between the two potential targets and, if so, whether this difference in magnitude coincided with a difference in temporal dynamics. Such differences in the temporal dynamics may include an earlier onset/steeper buildup and an earlier maximum of the effect of PROB manipulation on the movement direction compared with the effect of AMNT manipulation (see also Introduction; Suriya-Arunroj and Gail, 2015). To quantify the temporal dynamics of the influence of either prior, we conducted a time-continuous multiple-regression (TCMR) analysis (Scherbaum et al., 2010; Scherbaum and Dshemuchadse, 2020; see also Dotan et al., 2019).

In brief (see below for a detailed description including the necessary preprocessing steps), we fitted a series of linear regression models with the PROB and AMNT manipulations as predictors to the movement direction at multiple, densely sampled time points along the movement. We then concatenated the resulting regression coefficients to separate TCMR curves for PROB and AMNT and analyzed these TCMR curves in a fourfold fashion. First, we separately tested the PROB and AMNT TCMR curves for significance against zero to establish the individual time course of the effect on the movement of each prior. Second, we subtracted the AMNT TCMR curve from the PROB TCMR curve and tested this difference curve against zero to assess during which parts of the movement the two priors differed in their magnitude of effect. Third, we normalized each individual curve to a maximum of 1 and again tested the difference between the now normalized PROB and AMNT TCMR curves against 0. We did this to assess whether the effect of one prior increased more steeply than the other independent of the overall magnitude of the influence of the effect on the slope of the TCMR curves. A steeper increase of the effect of the PROB prior compared with the effect of the AMNT prior would result in positive values in this normalized difference curve that occurred earlier than the peak in the corresponding raw PROB and AMNT TCMR curves. These first three analyses were conducted using cluster-based permutation (ClusP) tests (Maris and Oostenveld, 2007; see below for details) that allow for statistical testing of time series data while controlling for the multiple-comparison problem. Fourth, to account for between-participant differences in the TCMR curves, we extracted the peak size and time per curve, which we compared between PROB/AMNT priors using paired *t* tests. This allowed us to compare the peak impact of each prior on the movement while controlling for the possibility that these peaks occur at different times per participant and prior (which the ClusP test does not).

To apply the TCMR, we first normalized the movement direction data and rescaled the PROB/AMNT pre-cue levels as follows. Per trajectory sampling point t, we defined the (momentary) actual movement direction $\vec{d_{t}}$ as the direction of the vector from position $XY_{t}$ to $XY_{t+1}$. To normalize the movement direction to continuous values between −1 and +1, we then determined (again per sampling point) the range of all potential movement directions aimed (1) anywhere at the later chosen target (+1) and (2) anywhere at the later unchosen target (−1). Momentary movement directions ranging between these two extremes resulted in intermediate values, according to the following formula:

$$\text{Normalized movement direction}=-2\times \left(\frac{{\text{θ}}_{a}}{{\text{θ}}_{b}}-0.5\right).$$

**Figure 2.**
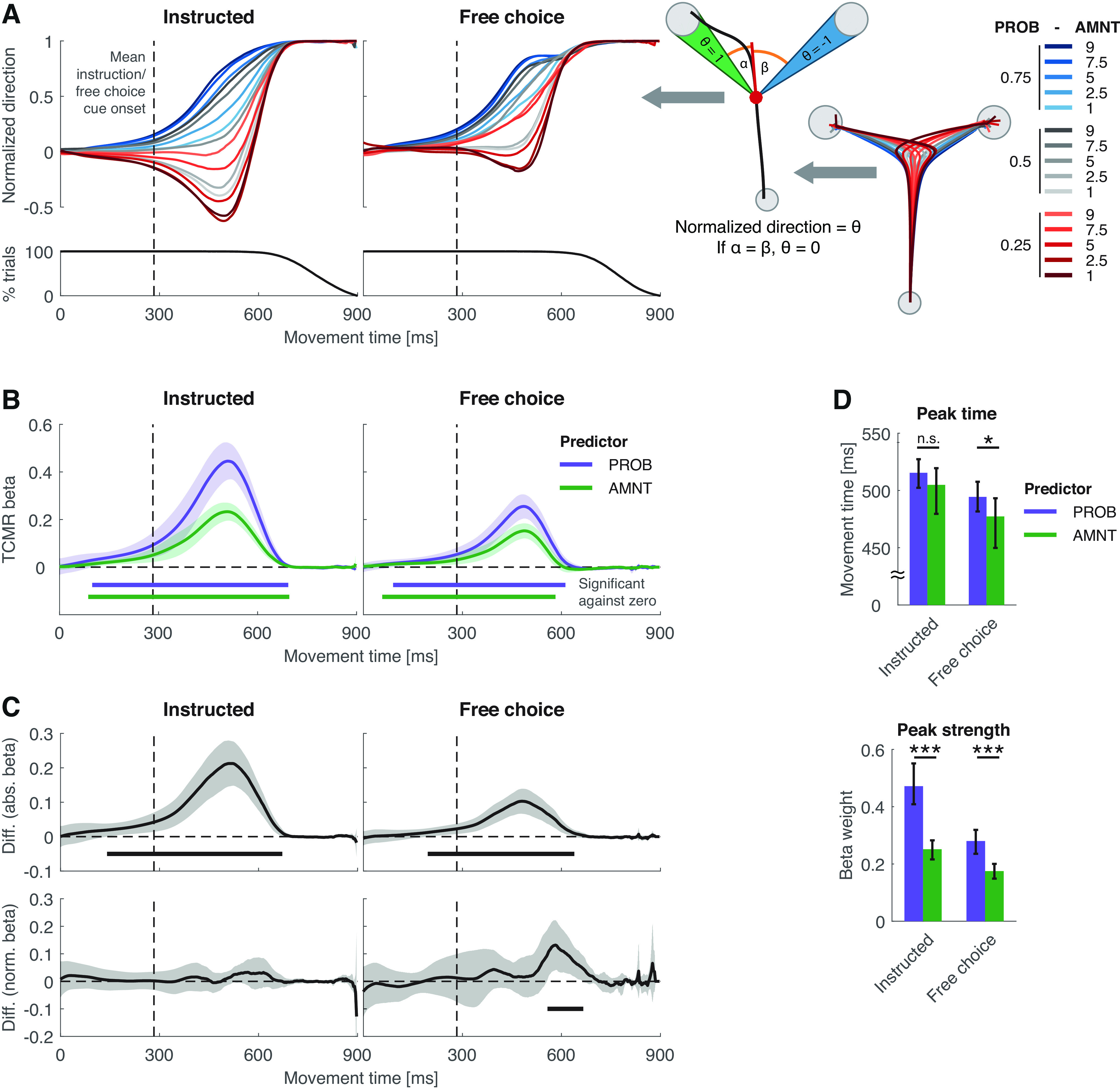
Time-continuous multiple regression results. ***A***, Left, center, Grand average normalized direction (1 = aimed at chosen target) as a function of movement time (i.e., the time elapsed since movement initiation, colored top curves; Extended Data Fig. 2-1, per-participant data) and average per-subject percentage of trials for which there is data at each given movement time stamp (black curve at bottom). Right, Computation of the normalized movement direction from the trajectories (see also Materials and Methods). ***B***, PROB and AMNT TCMR β weights. Horizontal bars represent movement time segments at which these β weights were significantly different from zero (determined via ClusP test; α = 0.05). ***C***, Top, Mean per-participant difference between the PROB and AMNT β curves from ***B***. Bottom, Same difference but after normalizing the per-participant curves to peak strength = 1. Horizontal bars represent a significant difference from zero as in ***B***. Error bands in ***B*** and ***C*** represent the 95% confidence intervals of the mean (Extended Data Table 2-1, full ClusP test results for ***B*** and ***C***). ***D***, Mean ± bootstrapped (*N* = 2000) 95% confidence intervals of the per-subject TCMR β curve peak times (in terms of time elapsed since movement initiation) and peak strength. n.s., Not significant. *p < 0.05, **p < 0.01, ***p < 0.001 for paired *t* tests. Extended Data Figure 2-2, per-participant data underlying ***B–D***, Exentended Data Figure 2-3, supplementary peak time and peak strength comparisons between PROB and AMNT, Extended Data Table 2-2, full peak time and peak strength comparison results for ***D*** and Extended Data Figure 2-3.

$\theta_{a}$ refers to the angle between $\vec{d_{t}}$ and $\vec{d_{c}}$, where $\vec{d_{c}}$ shares its origin with $\vec{d_{t}}$ and is directed such that it is aimed at the later chosen target while simultaneously minimizing $\theta_{a}$ (i.e., $\vec{d_{c}}$ is the closest hypothetical movement direction aimed from $XY_{t}$ to the target). $\theta_{b}$ refers to the angle between $\vec{d_{c}}$ and $\vec{d_{u}}$, where $\vec{d_{u}}$ is computed like $\vec{d_{c}}$ but with respect to the later unchosen target. This means, when the difference between the actual movement direction and the closest direction aimed at the later chosen target was equal to the difference between the actual movement direction and the closest direction aimed at the later unchosen target, the normalized movement direction was zero (Fig. 2*A*). By normalizing the movement direction, we were able to study the extent to which a participant leaned toward one target over the other independent of the cursor position relative to each respective target. Put differently, without this normalization, orienting the movement direction [e.g., 25% toward a target (between the vertical and the closest in-target direction at the current cursor location)] translates into larger movement direction angles (again, measured from the vertical) as the movement progresses along the vertical and therefore would falsely indicate a higher degree of commitment toward this target.

10.1523/ENEURO.0240-22.2023.f2-1 Figure 2-1 Per-participant normalized direction–movement time curves. Normalized direction (1 = aimed at chosen target) as function of movement time (i.e., the time elapsed since movement initiation), corresponding to Figure 2*A*. Each line represents the cross-trial average of a single participant (*N* = 20; all participants are included in this figure). Download Figure 2-1, EPS file.

10.1523/ENEURO.0240-22.2023.f2-2 Figure 2-2 Per-participant TCMR results. Each line represents a single participant (*N* = 20). ***A***, TCMR β curves (corresponding to Fig. 2*B*). ***B***, Differences between absolute PROB and AMNT TCMR β curves (corresponding to Fig. 2*C*, top). ***C***, Differences between normalized PROB and AMNT TCMR β curves (corresponding to Fig. 2*C*, bottom). ***D***, TCMR onset times, peak times, and peak strengths as in Figure 2*D*. Bar plots represent cross-participant mean values; line plots represent individual participant data. Download Figure 2-2, EPS file.

**Figure 3:**
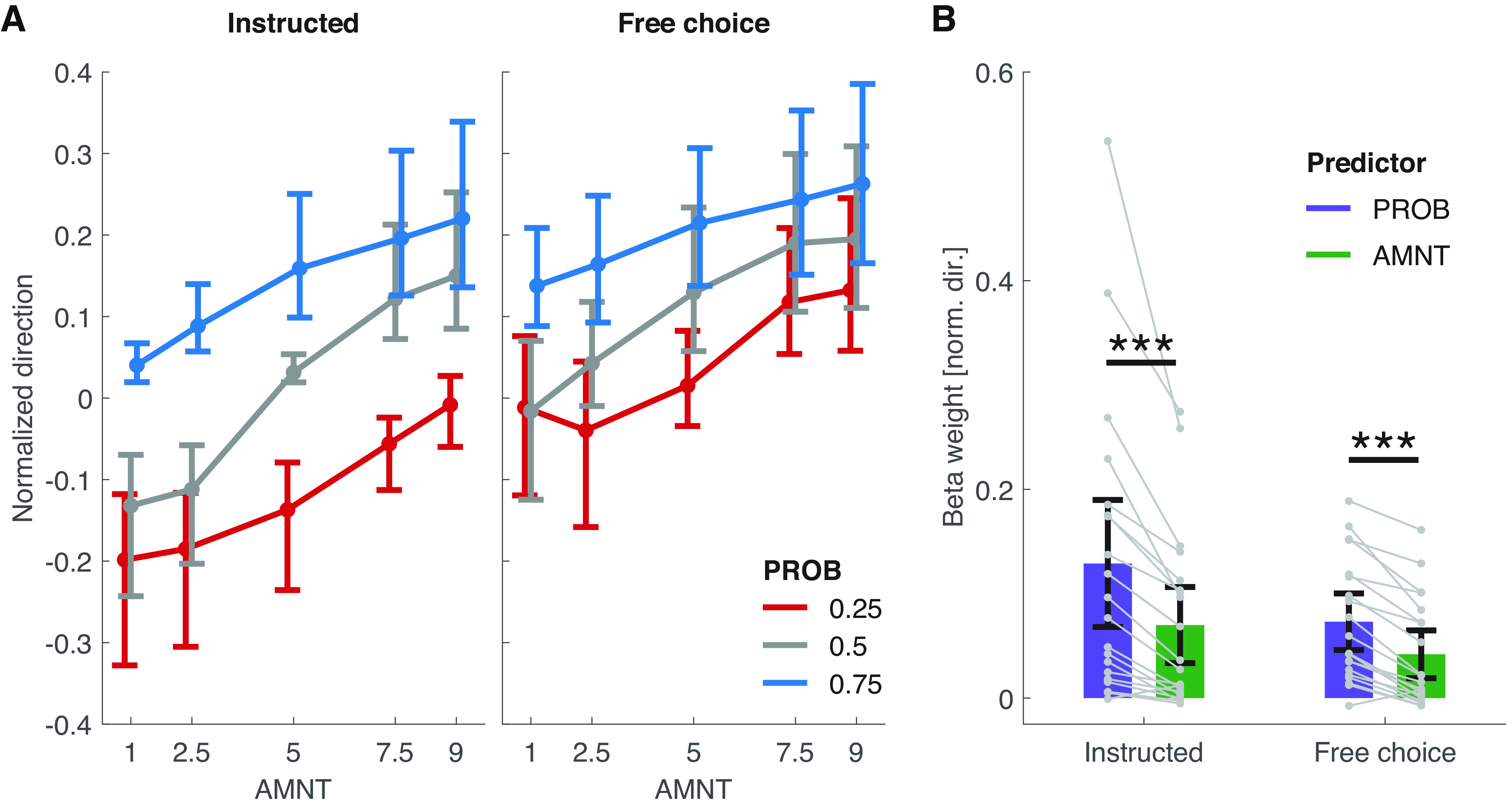
Early biases. ***A***, Total mean ± bootstrapped (*N* = 2000) 95% confidence intervals of the per-participant mean values of the early biases (normalized movement direction 50 ms post-instruction/free-choice cue onset). Extended Data Figure 3-1, per-participant data. ***B***, β weights resulting from fitting M2 to the data from ***A***. Bars and error bars represent the fixed effects of PROB and AMNT and their 95% confidence intervals; gray points and lines represent the per-subject random effects of PROB and AMNT. Significance marker conventions are as in Figure 2*D* (Extended Data Table 3-1, full M2 results).

10.1523/ENEURO.0240-22.2023.f2-3 Figure 2-3 Session-wise TCMR peak strengths and peak times. Across all rows, left to right: per trial-type comparison of PROB and AMNT peak times, and peak strengths. Bar plots and error bars represent cross-participant mean values and bootstrapped (*N* = 2,000) 95% confidence intervals of the mean; line plots represent individual participant data (*N* = 20). Significance marker conventions are as in Figure 2*D* (Extended Data Table 2-2, *t*-values and *p*-values). ***A***, Comparison of PROB and AMNT results in the session in which PROB was cued first. ***B***, Comparison of PROB and AMNT results in the session in which AMNT was cued first. ***C***, Comparison of PROB results from the session in which PROB was cued first with AMNT results from the session in which AMNT was cued first. ***D***, As in ***C***, but with both PROB and AMNT cued second. Download Figure 2-3, EPS file.

10.1523/ENEURO.0240-22.2023.tab2-1 Table 2-1 ClusP test statistics for Figure 2 *B-C*. *t*-Mean_original_ and *p*-values of the significant clusters displayed in Figure 2, *B* and *C*. *t*-mean_original_ refers to the arithmetic mean over all single-time point *t*-values that make up the *t*-sum_original_ of the significant cluster. The *p*-values that correspond to a *t*-mean_original_ are computed as the percentile of the *t*-sum_original_ with respect to the null distribution (see Materials and Methods). Download Table 2-1, DOCX file.

10.1523/ENEURO.0240-22.2023.tab2-2 Table 2-2 *t*-Test statistics for Figure 2*D* and Extended Data Figure 2-3. *t*-Test statistics for the pairwise PROB versus AMNT comparisons of TCMR peak time and peak strength. EDF, Extended data figure. All df value = 19. Download Table 2-2, DOCX file.

To directly compare the magnitude of the PROB and AMNT regression coefficients, both predictors were centered and rescaled before entering them into the regression analysis. We centered PROB and AMNT to their respective baseline levels (i.e., by subtracting 0.5 from all PROB levels and 5 from all AMNT levels). The centered PROB levels were rescaled by dividing them by 0.25, while the centered AMNT levels were rescaled by dividing them by 2.5. Therefore, PROB was centered and rescaled to 0.25 = −1, 0.5 = 0, and 0.75 = 1; and AMNT to 1 = −1.6, 2.5 = −1, 5 = 0, 7.5 = 1, and 9 = 1.6. The resulting regression weights express the change in the response variable relative to the PROB and AMNT baseline conditions (0.5 and 5, respectively), and ±50% changes (i.e., ±0.25 PROB/±2.5 AMNT) from the baseline are scaled to 1 in both predictors.

Ultimately, we fitted, per participant and interpolated time point *t*, linear model M1 (see below) to the normalized movement direction, using the rescaled PROB and AMNT levels as predictors (this and all forthcoming linear models are described using Wilkinson notation and include an intercept even if not spelled out explicitly). The resulting regression weights per time point were combined to time-continuous regression curves per participant and predictor. These TCMR curves quantify the temporal evolution of the influence of PROB and AMNT priors on the movement direction, as follows:

$$\text{M1: }{\text{Normalized direction}}_{\text{Interpolated time point t}}∼{\text{ PROB}}_{\text{rescaled}}+{\text{ AMNT}}_{\text{rescaled}}.$$

We tested the PROB and AMNT TCMR curves and the difference between these curves for significance against zero (now on the cross-participant level instead of the per-participant level) using ClusP tests [Maris and Oostenveld, 2007 (implementation was based on the study by Dann et al., 2016)] to account for multiple comparisons arising from having to test at up to 301 time points (see data preprocessing above). The ClusP tests were applied as follows. Per time point, a paired *t* test was performed where one sample consisted of the PROB, AMNT, or PROB-AMNT difference (see above) TCMR β weights obtained for this time point (sample 1), while the other sample consisted of an identical number of zeros (sample 2). Clusters were defined as temporally adjacent time points with significant *t* test results (α = 0.05) and an identical direction of effect. Per cluster, the *t*-values were accumulated (*t*-sum_original_). Next, this procedure was repeated 100,000 times with permutation. Here, on each iteration, the assignment of the TCMR β weights and the zeros to samples 1 and 2 was randomly permutated per participant. Again, *t*-sums were computed for each cluster (*t*-sum_permuted_). The largest *t*-sums_permuted_ of each iteration were combined to a null distribution of chance level largest-cluster *t*-sums. The original clusters were deemed statistically significant at α = 0.05 if their *t*-sum_original_ was >95% of the values of the null distribution. Therefore, statistical inference was based on the cluster level, not the level of the individual per-time point *t* tests. For each significant cluster, we report the arithmetic mean of the *t*-sum_original_ as test statistic and the percentile of *t*-sum_original_ with respect to the aforementioned null distribution as a *p*-value. We do not report the *t*-sum_original_ itself as a test statistic since this metric depends not only on the magnitude of effect but also the width of the cluster.

#### Early movement biases and time points of overt commitment

The TCMR analysis combines all levels of PROB and AMNT to compute a single estimate (per time point) of the strength of each prior on the movement. Consequently, the TCMR analysis does not allow for comparing single PROB/AMNT conditions (e.g., the PROB/AMNT 0.75/2.5 and 0.25/7.5 conditions). These conditions are matched in their expected value but have either a larger PROB or AMNT value than the alternative option. They thus allow to determine the influence of expected value (both PROB and AMNT weighted equally) versus a dominating influence of PROB over AMNT (or vice versa) on the movement. To this end, we computed two measures on a per-trial basis. First, we computed the early bias, which is the normalized movement direction 50 ms post-instruction cue onset. We chose this time point late enough in the trial for biases to become visible, but early enough that participants were not yet able to respond to the instruction/free-choice cue (the minimum delay to which motor corrections in response to visual stimuli can occur is ∼110 ms; Brenner and Smeets, 1997; Carroll et al., 2019). The early bias thus serves as a measure of the influence of the PROB and AMNT pre-cues on the movement trajectory.

Second, we computed the time points of overt commitment [i.e., times of commitment (TOCs); Ulbrich and Gail, 2021] as estimate for the mid-movement decision time. In brief, the TOC is determined, per trial, as the point from which on the difference between the actual movement direction and the closest direction aimed at the later chosen target starts to monotonically decrease until the actual direction remains aimed at the target for the rest of the movement. Importantly, this means that the TOC is not influenced by the magnitude of movement direction adjustment to aim at the target, but instead recovers the point at which the adjustment is initiated. We implemented the cone method using the code provided in the study by Ulbrich and Gail (2021) and applied the tolerance criterion (set to 3°), the overshoot criterion, but not the speed criterion.

To determine the relative influence of the PROB and AMNT priors on both the early bias and TOCs, we fitted the generalized linear mixed-effects (GLME) model (MATLAB function fitglme) M2 separately to each measure and separately to instructed and free-choice trials (i.e., four variants of M2 in total), using the rescaled PROB and AMNT pre-cue levels described above and adding a random intercept and random slopes per participant (for this and all forthcoming models, random effects are specified in parentheses following the fixed effects, and random intercepts are always included even if not spelled out explicitly in the model), as follows:

$$\text{M2: }{\text{early bias or TOC ∼ PROB}}_{\text{rescaled}}{\text{ + AMNT}}_{\text{rescaled}}\text{+ }\left({\text{PROB}}_{\text{rescaled}}{\text{ + AMNT}}_{\text{rescaled}}\text{| participant}\right).$$

We additionally performed pairwise comparisons of selected PROB/AMNT conditions to quantify differences in early bias and TOC between conditions with identical EV. Specifically, we compared the early biases and TOCs between (1) the PROB/AMNT 0.75/2.5 versus 0.25/7.5 condition (see above) and (2) the 0.75/1 versus 0.25/9 condition.

We did not include PROB × AMNT (i.e., expected value) interaction terms in models M1 and M2 as the results and post-experiment surveying of the participants suggested a nonlinear PROB × AMNT interaction. Specifically, the effect of PROB on early bias (and, by extension, the full trajectories as modeled by M1) and TOC appeared largest when AMNT was equal across targets, and vice versa. The inclusion of a PROB × AMNT interaction term in our model does not capture this nonlinear pattern well (<1% increase of explained variance; data not shown). We did not opt for more complex modeling to capture this observation a posteriori, since the exact interaction pattern was not part of our original research question.

#### Choice proportions

We asked how the PROB and AMNT priors affected the value-neutral free-choice proportions (CP), both in general and depending on the prior-induced variability in movement direction before choice. To investigate the overall choice preferences, we assessed how the proportion of choosing the high-PROB (0.75) target over the low-PROB (0.25) target depended on the AMNT level associated with the high-PROB target [AMNT_High-PROB_, logistic GLME M3 [see below)]. Here, we subtracted 1 from each AMNT level to let the intercept of M3 reflect the PROB = 0.75 CP at the lowest possible AMNT_High-PROB_ level. Note that M3 already includes most high-AMNT (7.5/9) CPs either because they are tied to high-PROB CPs (PROB/AMNT = 0.75/7.5 and 0.75/9) or are equal to 1 minus a high-PROB CP (PROB/AMNT = 0.25/7.5 and 0.25/9). Therefore, we restricted the statistical analysis of the high-AMNT CPs (logistic GLME M4; see below) to testing how the high-AMNT CPs depended on the high-AMNT level (AMNT_High_; AMNT = 7.5/9, rescaled to −1/1) in PROB = 0.5:0.5 trials. By rescaling AMNT_High_, the intercept of M4 reflects the average proportion of choosing any of the two high-AMNT options in PROB baseline trials, as follows:

$$\text{M3: }\text{High}-\text{PROB chosen }∼{\text{ AMNT}}_{\text{High}-\text{PROB}}+\left({\text{AMNT}}_{\text{High}-\text{PROB}}|\text{ participant}\right),$$

$$\text{M4: }\text{High}-\text{AMNT chosen }∼{\text{ AMNT}}_{\text{High}}+\left({\text{AMNT}}_{\text{High}}|\text{ participant}\right).$$

Since participants were able to gradually direct their movements toward one target at the expense of the alternative target before the instruction/free-choice cue was displayed, we also assessed how such an early bias in movement direction systematically covaried with the subsequent choice. Note that, here, we do not imply any causal direction between these two phenomena. An early bias toward a target may on the one hand be the premature expression to choose a target once allowed to do so. On the other hand, such an early bias may also incentivize participants to then choose this target since the biomechanical loss of following through with the early directional tendency is lower than redirecting the movement toward the alternative target. We statistically assessed how early biases toward one target over the other coincided with congruent tendencies to then choose these targets separately for each condition included in M3 and M4. To this end, we fitted the logistic GLME M5 (see below) to either the high-PROB CPs (for each condition included in M3) or the high-AMNT CPs (for each condition included in M4), using the normalized movement direction measured 50 ms post-instruction/free-choice cue onset as predictor that represented the early bias. Here, the normalized movement direction was recoded such that 1 corresponds to aiming the movement at the high-PROB target (when modeling the high-PROB CPs) and the high-AMNT target (when modeling the high-AMNT CPs), respectively, while −1 now corresponds to aiming at the low-PROB/AMNT target. To investigate the role of biomechanical loss on choice independent from the PROB/AMNT manipulations, we also fitted M5 to the proportion of right-hand choices in PROB/AMNT = 0.5:0.5/5:5 trials (early bias = 1, aiming at right-hand target; early bias = −1, aiming at leftward target), as follows:

M5:  High-PROB chosen OR high-AMNT chosen OR right-hand target chosen∼ early bias  + (early bias | participant).

## Results

We conducted a go-before-you-know experiment in which participants performed reach movements towards one of two potential targets with varied prior instruction probabilities (PROB) and prior reward amounts (AMNT). In two-thirds of all trials, subjects were instructed to reach to a specific target, which was selected according to the PROB prior and rewarded according to the AMNT prior, while in the remaining third of randomly interspersed trials, participants freely decided between the two, then equally unrewarded, targets. We investigated the time course and magnitude of the influence of PROB and AMNT priors on movement direction, the effect of the priors on the time to commitment to a target, and the effect of the priors and the interim movement direction on choice preferences. We hypothesized that the PROB prior has a larger influence on movement and choice than the AMNT prior and asked whether this difference in magnitude is accompanied by an earlier onset of the PROB effect.

### Larger influence of PROB than AMNT on movement direction despite near-identical time course

The normalized movement direction curves show that both the PROB and AMNT priors impacted the participants’ movements (Fig. 2*A*, Extended Data Fig. 2-1). In instructed trials, participants began to gradually direct their movements toward the PROB = 0.75 target early in the movement (i.e., before the instruction/free-choice cue was shown; Fig. 2*A*, positive Y values of the blue curves to the left of the dashed vertical line). This bias was amplified or attenuated, depending on whether the AMNT associated with the PROB = 0.75 option was high or low (Fig. 2*A*, fanning out of the blue curves). The AMNT manipulation was also able to elicit a directional bias on its own [i.e., when the PROB pre-cue was balanced; Fig. 2*A*, fanning-out of the gray (PROB = 0.5) curves]. Note that between movement start and instruction cue onset, the PROB = 0.75 (blue) curves and PROB = 0.25 (red) curves conceptually are mirror images of one another, while still comprising complementary subsets of trials. Whenever, within a trial, one target was associated with a PROB and AMNT of (e.g., 0.75 and 9), the other target was associated with a PROB and AMNT of 0.25 and 1. Therefore, a consistent (across-trial) preinstruction onset bias toward the 0.75/9 target appeared as a symmetrical bias toward the instructed target (if 0.75/9 was instructed) and against the instructed target (if 0.25/1 was instructed), respectively. The same rationale applies to the gray curves (PROB/AMNT 0.5/9 vs 0.5/1, and 0.5/7.5 vs 0.5/2.5, respectively).

In free-choice trials, during the early portion of the movements, participants were similarly biased toward high-PROB/AMNT options, but showed little to no bias away from the later chosen target (Fig. 2*A*, lack in early downward deflections of the curves). This was to be expected, since here participants often selected the target that was already aligned with their early bias (resulting in positive Y values in the PROB = 0.75 curves) and selected PROB = 0.25 options predominantly when they were not biased toward either target (resulting in Y values that were relatively closer to zero in the red curves). Selecting the closer target during free choices most likely reflected an ergonomic consideration as there was no AMNT-related benefit of one target over the other anymore. This discrepancy between instructed and free-choice trials also accounts for the relatively smaller effects of PROB and AMNT in free-choice compared with instructed trials in the forthcoming analyses and is therefore not examined any further.

We applied a TCMR analysis to the data shown in Figure 2*A* to quantify the temporal evolution of the PROB and AMNT prior effects on the decision process as observed in the time course of the movement direction. To improve legibility, all statements in the following paragraphs within this section apply to both instructed and free-choice trials unless noted otherwise, and the corresponding statistics and single-participant data are provided in Extended Data Table 2-1 and Table 2-2 and Extended Data Figure 2-2, respectively.

Congruent with the raw data pattern, both, PROB and AMNT priors affected the movement direction over a large portion of the movement, with participants incorporating the PROB and AMNT information into their movements already well before the time of the instruction/free-choice cue (i.e., before knowing which target or targets they were supposed to/allowed to acquire; Fig. 2*B*, Extended Data Table 2-1). In line with our hypothesis, the PROB prior had a consistently larger effect than the AMNT prior across a large portion of the movement (where the difference between the PROB and AMNT TCMR curves was significantly greater than zero; Fig. 2*C*, top, Extended Data Table 2-1) as well as in the isolated peak effects (Fig. 2*D*, peak strength, Extended Data Table 2-2). In other words, the participants’ tendency to gradually direct their movement toward a target depended to a larger extent on whether this target was associated with a higher PROB level than it did on whether it was associated with a higher AMNT level (both compared with the PROB/AMNT levels of the alternative target).

We then asked whether the larger effect of the PROB prior on the movements was accompanied by a relatively faster unfolding of this effect. We tested for such a faster unfolding in a threefold manner. However, contrary to our hypothesis, we did not find any evidence of a faster time course of the effect of the PROB prior. First, we assessed whether the difference between PROB and AMNT during the buildup of their effects on the movements (i.e., along the rising slope left of the peaks; Fig. 2*B*) reflected a faster rising of the PROB effect or simply emerged from the overall larger magnitude of the effect of PROB prior. To remove these differences in PROB–AMNT scaling, we normalized all TCMR curves to a peak value of 1 and subtracted the normalized AMNT curves from the normalized PROB curves. A faster unfolding (i.e., a steeper rise) of the PROB effect compared with AMNT would then show in the resulting curve as a positive deviation from zero during the prepeak movement epoch (i.e., earlier than ∼500 ms). This was not the case, and, instead, we only found a spurious significant deviation on the postpeak downward slope in free-choice trials (Fig. 2*C*, bottom, Extended Data Table 2-1). Second, the peak time did not significantly differ between the PROB and AMNT effects in instructed trials (mean difference, 11 ms; *p *=* *0.10) and only marginally differed in free-choice trials (mean difference, 17 ms; *p *=* *0.02; Fig. 2*D*, Extended Data Table 2-2).

In summary, our TCMR analysis shows that participants took both the PROB and the AMNT priors into account when guiding their movements and the putatively underlying choice process. These effects emerged early and were measured across most of the movement period with the exception of only the latest stage, where, because of its calculation, the normalized movement direction converged to one. We hypothesized that these effects were larger for PROB than AMNT and that this difference in magnitude was accompanied by an earlier onset/faster unfolding of the effect of the PROB prior. While we were able to confirm such a larger influence of a PROB prior on movement direction, the time courses of effect were markedly similar between PROB and AMNT.

As previously mentioned, each participant completed two main sessions, one with the PROB prior being cued first, the other with the AMNT prior being cued first. We pooled the data across sessions to study the effects of each prior independent of cueing order. To rule out that this data pooling decreased the sensitivity to detect temporal differences between the effects of two priors, we repeated the analyses shown in Figure 2*D* (comparison of peak time and peak strength between PROB and AMNT) on different single-session levels (Extended Data Fig. 2-3, Extended Data Table 2-2). In the sessions where the PROB prior was cued first (i.e., cued block-wise), the PROB peak emerged before the AMNT peak, and vice versa for the sessions in which AMNT was cued first. This cueing order dependence was intentionally avoided by pooling across sessions. All other comparisons shown in Extended Data Figure 2-3 were markedly similar to the results shown in Figure 2*D*.

### Larger influence of PROB than AMNT on early biases in movement direction and the time point of overt commitment

To study the influence of PROB and AMNT in single conditions, we extracted the early bias (i.e., the normalized movement direction 50 ms post-instruction cue onset), and the time participants needed to commit to a target (TOC). The TOC is not equivalent to the time participants needed to fully acquire a target but rather measures the time participants committed to a target mid-flight (i.e., started to unequivocally adjust their movement toward the later acquired target; Ulbrich and Gail, 2021). Measuring the TOC allows better (compared with the TCMR analysis) comparison of our results to more conventional “decide-then-act” studies, where scalar reaction times are used as a measure for choice latency.

**Figure 4.**
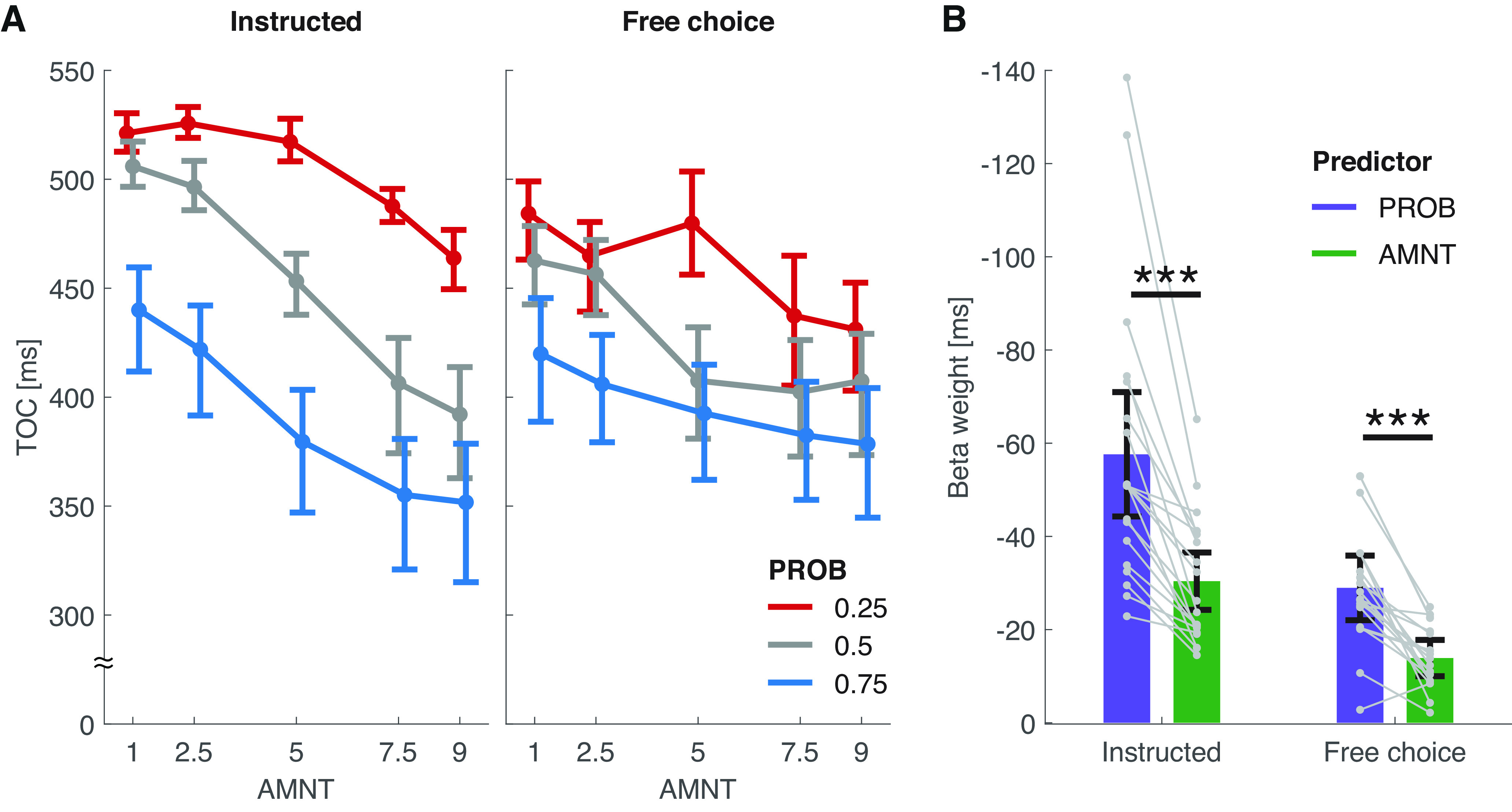
Time points of overt commitment. ***A***, Total mean ± bootstrapped (*N* = 2000) 95% confidence intervals of the per-participant mean values of the TOCs (Extended Data Fig. 4-1, per-participant data). ***B***, β weights resulting from fitting M2 to the data from ***A***. Bars and error bars represent the fixed effects of PROB and AMNT and their 95% confidence intervals; gray points and lines represent the per-subject random effects of PROB and AMNT. Significance marker conventions are as in Figure 2*D* (Extended Data Table 4-1, full M2 results).

In both instructed and free-choice trials, participants moved more strongly toward targets associated with a high-PROB prior compared with low-PROB targets (early bias; Fig. 3*A*, vertical spread between the separate curves; M2: instructed: β = 0.13, *p *<* *0.001; free-choice: β = 0.07, *p* < 0.001) and committed earlier to high-PROB compared with low-PROB targets (TOC; Fig. 4*A*, vertical spread between the separate curves; M2: instructed: β = −57.62, *p *<* *0.001; free-choice: β = −28.95, *p* < 0.001). We observed a similar pattern for high-AMNT targets compared with low-AMNT targets (Figs. 3*A*, 4*A*, vertical spread within each curve; M2 on the early bias data: instructed: β = 0.07, *p *<* *0.001; free-choice: β = 0.04, *p *<* *.* *001; M2 on the TOC data: instructed: β = −30.38, *p *<* *0.001; free-choice: β = −13.89, *p* < 0.001; Extended Data Figs. 3-1, 4-1, per-participant early bias/TOC traces, Extended Data Table 3-1, Table 4-1, full early bias/TOC M2 results). In line with the previously described TCMR results, this effect was larger for PROB than for AMNT in both trial types, both for the early bias metric (Fig. 3*B*; paired *t* test between single-participant random effects of PROB and AMNT: instructed:v*t*_(19)_ = 4.11, *p *<* *0.001; free-choice: *t*_(19)_ = 7.18, *p *<* *0.001) and the TOC metric (Fig. 3*B*; instructed: *t*_(19)_ = −5.82, *p *<* *0.001; free-choice: *t*_(19)_ = −6.45, *p *<* *0.001).

10.1523/ENEURO.0240-22.2023.f3-1 Figure 3-1 Per-participant early biases. Per-participant (*N* = 20) mean early biases (normalized movement direction 50 ms post-instruction/free-choice cue onset), corresponding to Figure 3*A*. Missing data points in the top right panel result from a single participant never having selected the PROB/AMNT = 0.25/1 and 0.25/5 options in free-choice trials. Download Figure 3-1, EPS file.

10.1523/ENEURO.0240-22.2023.tab3-1 Table 3-1 M2 results (early bias). Results of the GLME M2 fitted onto the early bias data. CI, Confidence interval; LB, lower boundary; UB, upper boundary. Separate models were computed for each trial type. Download Table 3-1, DOCX file.

Additional evidence for the dominating influence of the PROB prior over the AMNT prior can be found in pairwise comparisons of PROB–AMNT combinations that were either matched in EV (PROB/AMNT = 0.75/2.5 vs 0.25/7.5) or had a higher EV in favor of the low-PROB/high-AMNT option (PROB/AMNT = 0.75/1 vs 0.25/9). If participants had weighted the movement targets strictly according to their EV instead of weighting PROB more than AMNT, early bias TOCs should have been similar between the PROB/AMNT 0.75/2.5 and 0.25/7.5 conditions, and larger in the PROB/AMNT 0.75/1 compared with the 0.25/9 condition. Instead, participants consistently moved more strongly toward the high-PROB targets and committed faster to them in the matched EV conditions (paired *t* test of the per-participant mean early biases: instructed: *t*_(19)_ = 3.53, *p *=* *0.002; free-choice: *t*_(19)_ = 2.90, *p *=* *0.009; TOCs: instructed: *t*_(19)_ = −5.68, *p *<* *0.001; free-choice: *t*_(19)_ = −3.42, *p *=* *0.003) and similarly strong/fast to the 0.75/1 and 0.25/9 targets despite their difference in EV in favor of the high-AMNT targets (early biases: instructed: *t*_(19)_ = 1.46, *p *=* *0.16; free-choice: *t*_(19)_ = 0.24, *p *=* *0.81; TOCs: instructed: *t*_(19)_ = −1.68, *p *=* *0.11; free-choice: *t*_(19)_ = −1.22, *p *=* *0.24).

10.1523/ENEURO.0240-22.2023.f4-1 Figure 4-1 Per-participant TOCs. Per-participant (*N* = 20) mean TOCs, corresponding to Figure 4*A*. Missing data points in the top right panel result from a single participant never having selected the PROB/AMNT = 0.25/1 and 0.25/5 options in free-choice trials. Download Figure 4-1, EPS file.

10.1523/ENEURO.0240-22.2023.tab4-1 Table 4-1 M2 results (TOC). Results of the GLME M2 fitted onto the TOC data. CI, Confidence interval; LB, lower boundary; UB, upper boundary. Separate models were computed for each trial type. Download Table 4-1, DOCX file.

### PROB and AMNT priors as well as biomechanics affect value-neutral free choice

**Figure 5.**
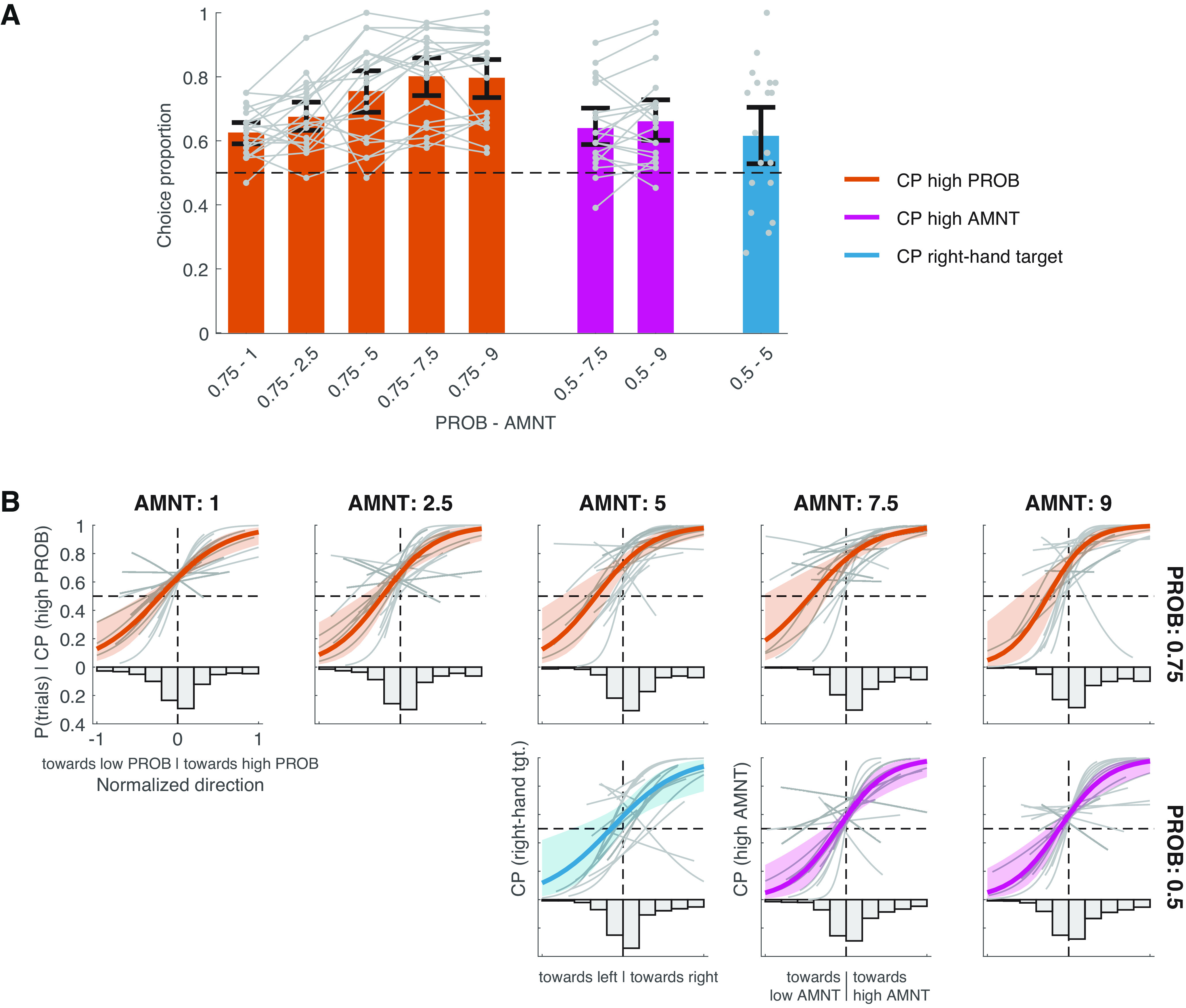
Choice preferences. ***A***, Orange, mean per-participant proportions of choosing the high-PROB (0.75) target over the low-PROB (0.25) target separately for each possible AMNT level associated with the high-PROB target. Magenta, Proportions of choosing the high-AMNT (7.5 or 9) target over the low-AMNT (2.5 or 1) target at PROB = 0.5. Blue, Proportions of choosing the right target in the PROB/AMNT = 0.5:0.5/5:5 baseline condition. Note that the proportions of choosing a high-AMNT target associated with PROB = 0.25 or PROB = 0.75 are included as part of the high-PROB choice proportions. The PROB/AMNT 0.25/7.5 and 0.25/9 choice proportions equal 1 minus the 0.75/2.5 and 0.75/1 choice proportions, respectively. Error bars are bootstrapped (*N* = 2000) 95% confidence intervals of the mean. Gray points and lines represent single-participant (*N* = 20) choice proportions (Extended Data Table 5-1, M3 and M4 results on the choice proportions displayed here). ***B***, Top, Proportions of choosing the high-PROB over the low-PROB target as a function of the normalized movement direction relative to the high/low-PROB target (1 = movement aimed at high-PROB target, −1 movement aimed at low-PROB target). Each panel represents one of the five possible AMNT values that were paired with the high-PROB target. The normalized direction was calculated 50 ms after the onset of the free-choice cue. Colored lines represent the marginal (i.e., fixed effects) M5 fits and their 95% confidence intervals. Gray lines represent the per-participant (*N* = 20) conditional (i.e., random effects) fits. Histograms represent the mean per-participant proportion of trials in each normalized direction bin (bin width, 0.2; for illustrative purpose only, M5 was fitted to the continuous normalized movement direction data). Bottom center, Proportion of right-hand choices as a function of the normalized movement direction (50 ms after instruction cue onset) relative to the right (normalized direction, 1) and left (normalized direction = −1) target in the PROB/AMNT = 0.5:0.5/5:5 baseline condition. Bottom right, Proportions of choosing the high-AMNT targets as a function of the normalized movement direction (50 ms after instruction cue onset) relative to the high-AMNT (normalized direction, 1) and low-AMNT (normalized direction, −1) targets (Extended Data Table 5-2, full M5 results).

Finally, we studied how the PROB and AMNT priors as well as early movement biases affected the value-neutral free-choice preferences (choice proportion, CP). In PROB = 0.25:0.75 trials, participants on average preferred the PROB = 0.75 target over the PROB = 0.25 target when the AMNT associated with the PROB = 0.75 target was only 1 (i.e., the preliminary EV of this target was lower than that of the alternative target; Fig. 5*A*; PROB – AMNT = 0.75–1; M3: intercept = 0.54, *p* ≤ 0.001). This CP bias in favor of the PROB = 0.75 target increased congruently with the AMNT level associated with this target (Fig. 5*A*, orange bars; M3: slope = 0.16, *p *<* *0.001; Extended Data Table 5-1, full M3 results). In PROB = 0.5:0.5 trials, the AMNT prior was able to elicit a choice bias of its own. Participants preferred high-AMNT over low-AMNT targets (Fig. 5*B*, magenta bars; M4: intercept = 0.68, *p *<* *0.001) but did not discriminate between AMNT = 7.5 and 9 (M4: slope = 0.06, *p *=* *0.22; Extended Data Table 5-1, full M4 results; see Materials and Methods for the reason that only PROB = 0.5 trials were included in M4). In trials where both PROB and AMNT were balanced across conditions (i.e., 0.5:0.5–5:5), participants on average exhibited a bias to choose the right-hand target (Fig. 5*B*, blue bar; *t* test against 0.5: *t*_(19)_ = 2.50, *p *=* *0.02).

10.1523/ENEURO.0240-22.2023.tab5-1 Table 5-1 M3 and M4 results. Results of the GLME M3 fitted onto the high-PROB choice proportions and M4 fitted onto the high-AMNT choice proportions. CI, Confidence interval; LB, lower boundary; UB, upper boundary. Download Table 5-1, DOCX file.

10.1523/ENEURO.0240-22.2023.tab5-2 Table 5-2 M5 results. Results of the GLME M5 fitted onto the per-PROB/AMNT condition choice probabilities as a function of the normalized movement direction. CI, Confidence interval; LB, lower boundary; UB, upper boundary. Download Table 5-2, DOCX file.

As shown above, participants gradually aimed their movements toward high-PROB and/or AMNT targets before they knew whether they were able to freely choose. We therefore examined to what extent the preference to freely choose high-PROB and/or AMNT targets covaried with these early biases in movement direction. To this end, we fitted M5 to the CP as a function of the normalized movement direction measured 50 ms after instruction cue onset (early bias) separately for each PROB–AMNT combination, as shown in Figure 5*A*. In all conditions where at least either PROB or AMNT was imbalanced across targets, the probability of choosing a target was highly linked to the extent to which the movement had been directed toward this target beforehand, regardless of whether the choice followed the overall CP shown in Figure 5*A* or not (Fig. 5*B*, M5, all slopes positive with *p *<* *0.001, Extended Data Table 5-2, full M5 results). Notably, participants still preferred the high-PROB and/or high-AMNT target congruent with the results shown in Figure 5*A* in trials with no overt early bias (M5, all intercepts >0 with *p *≤* *0.001). These two patterns partially extend to the CP for right-hand target choices in PROB/AMNT 0.5:0.5/5:5 targets where participants chose the right-hand target more often when they already exhibited an early bias toward it (slope, *p *=* *0.003) but did not significantly prefer this target in the absence of an early bias (intercept, *p *=* *0.08).

In summary, participants preferred high-PROB and high-AMNT targets over low-PROB/AMNT targets. In cases where the high PROB was associated with a low AMNT, the preference for high-PROB targets dominated the choice behavior. These overall choice preferences were further up-modulated and down-modulated by the participants’ early movement tendencies as an early bias toward a target increased the probability of subsequently choosing this target. When the PROB/AMNT priors were both balanced across targets, participants also predominantly chose the target they were biased toward early in the movement, indicating a desire to reduce biomechanical costs when selecting a target.

## Discussion

We asked how participants combine action PROB and associated reward AMNT priors to guide their reach movements and choices in a go-before-you-know (“online”) action selection task. By cueing the PROB and AMNT priors before movement onset and providing the choice-enabling instruction or free-choice cue mid-movement, the participants’ movement trajectories offered insight into the early dynamics of the effects of priors on the choice process before an informed commitment to a target was possible. To dissociate the influence of the priors PROB and AMNT from one another, we rewarded only instructed reaches according to the AMNT prior while free choices were always unrewarded (i.e., value neutral). In this way, the probability with which a target with a given reward was available was determined by the PROB pre-cue, but not the frequency with which this target was chosen in free-choice trials. As a result, participants aimed their movements toward targets associated with a high PROB and/or high AMNT (relative to the PROB and AMNT of the alternative target) before the instruction cue was provided and committed to these targets faster (TOC) in both instructed and free-choice trials. These effects were graded, but were dominated by PROB over AMNT priors to a degree that the preliminary expected value no longer explained the choice behavior well. Yet, the temporal dynamics with which the priors exerted their effect on movement trajectories were highly similar between both priors. We conclude that the action probability, probably because of the associated possibility of being able to plan the respective movement in advance, strongly dominates mid-movement decisions to the point that higher preferability of an action is traded against better plannability of the according movement.

The current study relates to previous works on the influence of prior probability and reward on action selection including both human online choice studies (Hudson et al., 2007; Chapman et al., 2010, 2015; Marti-Marca et al., 2020) and neurophysiological studies in monkeys (Pastor-Bernier and Cisek, 2011; Suriya-Arunroj and Gail, 2019). In these studies, only either prior probability or reward expectancy were manipulated. Therefore, while these studies in combination show that both prior probability and reward expectancy affect movement planning and early (precommitment) movement execution, they neither answer how strongly nor at which time course prior probability and reward expectancy affect movement and choice behavior relative to each other within the same decision. By simultaneously and orthogonally manipulating our PROB and AMNT priors in an online choice task, we specifically answer these questions.

Our combined PROB and AMNT prior manipulation revealed asymmetric effects of the two decision variables. The effect of action probability dominated behavior compared with the effect of preliminary expected reward in terms of early movement bias (graded orientation of the movement toward one target over the other before the onset of the instruction/free-choice cue), TOC, and free-choice proportions. Notably, while both PROB and AMNT priors affected movements in combination, the effect of AMNT modulation was, on average, not strong enough so that subjects would bias their early movements toward the low-PROB target. Also, TOC was modulated with AMNT prior, but no matter how high (within the limits of this study) the AMNT prior for the low-PROB target was, participants always committed to the high-PROB target faster. This means, strikingly, that even when the high-PROB target had a much lower preliminary EV (75% PROB × 1 AMNT = 0.75) than the low-PROB target (25% PROB × 9 AMNT = 2.25), participants on average still committed earlier to the high-PROB target when instructed/chosen. In a previous related study, biasing effects of AMNT priors where invariant to doubling of the reward amounts, including reward ratios of up to 12:0 (Suriya-Arunroj and Gail, 2015). Similarly, while CPs were biased in favor of both high-PROB and high-AMNT options in the current study, participants on average preferred only the high-AMNT option when it was associated with at least the same PROB as the low-AMNT option. The fact that the modulation of early bias and TOC by AMNT prior do not overlap in their ranges between low-PROB and high-PROB conditions comes with the observation that the effect of the PROB prior on the movements was largest when the AMNT prior was equal for both targets, and vice versa (Figs. 3, 4). Together with a seeming flooring and ceiling effect for early bias and TOC, this results in a nonlinear interaction between PROB and AMNT priors. Correspondingly, adding a PROB × AMNT interaction term to our linear modeling did not improve the explained variance (see Materials and Methods). As a conclusion, this pattern suggests a hierarchical selection process in which participants select the target with first priority according to the PROB prior, and only with subordinate priority according to the AMNT prior within each PROB condition, independent of the temporal order in which both priors were revealed to the participants.

The asymmetry between PROB and AMNT priors likely emerged from an increased behavioral relevance of the PROB prior compared with the AMNT prior for planning and controlling the target-associated movement. It is a valid strategy for participants in our task to prioritize the likely-to-be-instructed (“correct”) target, which was predictable to a certain degree via the PROB pre-cue, over the target with the highest payoff, since rewards were exclusively provided for instructed targets and participants could not influence which target was instructed. Only the PROB pre-cue helped to predict the correct movement, not the AMNT pre-cue, and preparing the correct movement made a successful completion of the movement under the given time and space constraints more likely. A different weighting of the PROB and AMNT priors may therefore reflect risk avoidance, although our task did not require subjects to make decisions under risk. In classical decision-making studies, risk refers to the probability of a negative outcome to occur once a choice is made (Kahneman and Tversky, 1979; Tversky and Kahneman, 1992). In the current study, rewards were deterministic but the movements themselves were risky as participants were able to fail a trial, for example, by accidentally moving into the uninstructed target or not reaching the correct target in time, which, in the case of instructed trials, leads to missing out on the reward. Sensitivity to such motor risks has been demonstrated earlier (Trommershäuser et al., 2003a, b, 2006; Nagengast et al., 2010, 2011a, b; Carroll et al., 2019). Therefore, we attribute the strong dominance of PROB priors over AMNT priors to a strategy of preplanning the higher probability movement, which is reasonable for improving overall performance. Instead, biases toward the higher reward targets may be seen as “wishful thinking” and were most prominently visible in trials where the correct target was unpredictable (PROB = 0.5:0.5). In other words, the emerging plan for adjusting the movement based on an expected future required action (according to the more likely instruction) leads to visible bias in the early movement trajectory and, in case the instruction is omitted, a bias in value-neutral choices in favor of the originally more likely action, no matter how preferable the alternative action would have been.

While being asymmetric in amplitude, the effects of PROB and AMNT prior were synchronous in time. We did not expect this because of a previous study by Suriya-Arunroj and Gail (2015), in which PROB and AMNT priors were similarly paired with value-neutral free choices, but without a time-resolved analysis of how the effect of priors unfolds. The authors suggested that the PROB prior induces action planning in favor of the more likely target before the instruction/free-choice cue is provided, explaining why high-PROB targets lead to shorter reaction times when instructed and to faster and more frequent free choices in favor of this target. This interpretation is also supported by corresponding patterns of neural movement planning activity in the frontoparietal reach network of rhesus monkeys (Suriya-Arunroj and Gail, 2019). Conversely, the effects of AMNT prior in the study by Suriya-Arunroj and Gail (2015) were observed only in instructed trials, while reaction time benefits and choice biases were almost absent in free-choice trials. Accordingly, the AMNT prior was hypothesized to exercise its effect only once the instruction cue is provided and the final reward on successfully performing the reach is known. Following this rationale, reaction time benefits for reaching toward a high-AMNT target versus a low-AMNT target can only emerge in instructed trials and possibly reflect the motivational effects of being allowed to acquire a highly rewarded target versus being forced to acquire a low-rewarded target (see also Mir et al., 2011; Summerside et al., 2018). In contrast, our current results revealed that the effect of AMNT priors does indeed act on ongoing movements and choice behavior with a similar timeline as PROB priors, albeit with smaller effect strength, similar to what was previously suggested for baseline shifts in drift-diffusion models (Leite and Ratcliff, 2011; Mulder et al., 2012).

By asking our participants to initiate their movement before the instruction/free-choice cue was displayed, we allowed the PROB and AMNT priors to exercise their effect on behavior before the final target or targets and their associated rewards were known. Consequently, these effects may be viewed as “self-amplifying.” By biasing the movement toward a target early on, the distance and the biomechanical costs for turning toward this target continuously decrease compared with the alternative target as the movement progresses. Biomechanical effort is able to affect action choices independently of reward incentives (Cos et al., 2011, 2014; Morel et al., 2017) and discourages mid-movement changes of mind (Burk et al., 2014). Additionally, early biases are hypothesized to facilitate commitment to the bias-congruent target (Lepora and Pezzulo, 2015). Thus, even under a time-invariant influence of the priors on the decision process, allowing the priors to affect overt behavior magnifies their effect over the course of the movement. Requiring participants to withhold their movements until the instruction is displayed instead (Suriya-Arunroj and Gail, 2015), does not allow self-amplification, hence smaller effects such as the effect of the AMNT prior in free-choice trials may remain below the threshold of influencing overt behavior. Increased sensitivity might mark an advantage of online choice over reaction time paradigms (for review, see Freeman, 2018). More importantly, the mutual interdependence between planning and online control of movement and decision-making underscores the importance of sensorimotor contingencies and the action context in which decisions are situated (Lepora and Pezzulo, 2015; Hagura et al., 2017; Carsten et al., 2022).

Our task was not designed to answer the question of whether mid-target aiming in online choice paradigms is better explained by the averaging of parallel movement plans (Stewart et al., 2014; Gallivan et al., 2017) or the optimizing of single movement plans (Hudson et al., 2007; Haith et al., 2015; Nashed et al., 2017; Wong and Haith, 2017; Alhussein and Smith, 2021), and our results are compatible with both ideas. Early (precommitment) biases of movement direction toward one of two targets can be seen as imbalanced parallel movement planning toward both targets before movement onset (Stewart et al., 2014; Gallivan et al., 2017). Parallel planning that precedes a choice between action targets that had been observed before in dual-motor goal representations of the frontoparietal cortical networks of rhesus macaques (Cisek and Kalaska, 2005; Klaes et al., 2011; Suriya-Arunroj and Gail, 2019). The observed dominance of PROB priors over AMNT priors also complies with the alternative idea of strategic intermediate (i.e., initially aimed in between both targets with graded, PROB/AMNT-induced biases toward one target) trajectories. It was previously suggested that intermediate trajectories may stem from a single movement plan that optimizes task success (Hudson et al., 2007; Haith et al., 2015; Nashed et al., 2017; Wong and Haith, 2017; Alhussein and Smith, 2021). Biasing the early (preinstruction/free-choice cue) portion of the movements more strongly toward high-PROB than high-AMNT targets may have reflected such a desire to optimize task success, as discussed above.

Overall, our results demonstrate that the embodied go-before-you-know decision paradigms are a powerful approach to probing decision priors and integration of different decision variables in multiple-attribute decision-making. They further emphasize the tight interdependence between ongoing movement and choice in online decision-making, which is particularly relevant across species during natural behaviors like group foraging or chasing pray.

## Synthesis

Reviewing Editor: David Franklin, Technische Universitat Munchen Decisions are customarily a result of the Reviewing Editor and the peer reviewers coming together and discussing their recommendations until a consensus is reached. When revisions are invited, a fact-based synthesis statement explaining their decision and outlining what is needed to prepare a revision will be listed below. The following reviewer(s) agreed to reveal their identity: Joshua Cashaback.

Your manuscript has been reviewed by two experts in the field. They both have interest in the manuscript and think that this is an important question examining whether the amount of reward or the probability of reward has a stronger influence on the decision-making process during reaching movements. However, we all see significant issues. First, with the interpretation of the results - that probability is the dominant factor over reward amount – and how this relates to expected value. Second, how learning would influence these results, as extensive practice might be needed for developing priors. Thirdly we think that the manuscript would benefit from a more comprehensive comparison with past work in the literature. Nevertheless, there is sufficient interest to see a revised version of the paper. I encourage you to revise your manuscript, taking into account each of the concerns raised by the reviewers, and preparing a point-point-response.

General comments from the reviewers.

Here the authors asked the question of whether the probability of reward or the amount of reward had a larger influence on the unravelling decision-making process during online reaching movements. The results demonstrate that participants were able to adjust their movements online, once relevant information about the target became available. Overall, the manuscript is written well, and the question is important. However, there are three major concerns regarding the current version of the manuscript. First, the main interpretation is centred on the idea that probability is the dominant factor, over reward. However, it is unclear whether these results would hold if larger reward amounts were presented. The majority of interpretation and linear models ignore the interaction of probability and amount (i.e., expected value), which would likely explain the majority of the results and variance. There were instances where the expected value was constant across conditions, despite having different amount and probabilities. Highlighting comparisons between these conditions with more of the dependent measures (other than just TOC) would be particularly valuable. Second, is the issue of learning. It has been previously shown that (prior) information about the target position can be learnt across hundreds of trials during motor tasks. However, in the current task the probabilities of the binary target locations were visually communicated at the beginning of trials, but not really experienced by the participants over the relatively short blocks of 60-72 trials. Therefore, there are questions about whether the explicit probabilities stated here are meaningful for motor planning. Finally, the manuscript needs to be better positioned within the previous work in the field; highlighting similarities and differences with previous work in the literature.

Specific Comments.

Expected value: Central to the experimental question is whether one can partition and independently assess the influence of prior reward probability and prior reward amount on the unravelling decision-making process, which is expressed by the sensorimotor system via movement. Examining the data, it appears that the observed trends would largely be explained by expected reward (amount x probability; Fig. 2A). The authors dedicate a large amount of text in the introduction and methods about partitioning probability and amount priors (see comments below). This is further reflected in their statistical analyses where they include terms that independently relate to probability and amount. However, an interaction term of probability and amount would likely explain the majority of the variance in a linear model.

a. Can the authors also include additional analyses where they include an interaction term to M1 (line 335) and M2 (line 373) and interpret the subsequent results.

While the majority of analyses do not account for expected value, the authors did cleverly account for it in some locations. While perhaps it would have been a stronger experimental design to consistently control for expected value, there were specific conditions where the expected value was controlled and used by the authors to interpret the findings (lines 527-538: e.g., 0.75 x 5 (+ theta) vs 0.5 x 7.5 (+ theta) AND 0.25 (i.e. 75% right target) x 7.5 (- theta) vs 0.5 x 7.5 (-theta). These analyses are some of the most convincing to support the authors interpretation. This was performed for the TOC analyses only.

b. It would be very beneficial if the authors could perform a further analysis, for the particular conditions where expected value is controlled (e.g., see directly above), when assessing initial movements. Can the authors please examine normalized movement direction for these conditions [0.75 x 5 (+ theta) vs 0.5 x 7.5 (+ theta) AND 0.25 (i.e. 75% right target) x 7.5 (- theta) vs 0.5 x 7.5 (-theta)] prior to the cue of instructed vs free choice trial. It would also be beneficial to include these results within the main figures and highlighted within the results section of the manuscript.

When reading the paper, it was difficult to parse how reward amount would influence reward probability. The introduction states (lines 67-69), “...we made the probability of obtaining a reward independent from the AMNT prior by combining rewarded instructed trials with value-neutral (i.e., unrewarded) free-choice trials...”. When reading the paper linearly (abstract -> introduction -> methods, etc) this sentence was unclear. Further, in the methods (lines 205-226) the authors dedicate substantial text to the same idea, but the idea is still difficult to parse. A numerical example, or equation, would greatly benefit in guiding the reader through the idea the authors are trying to highlight. That mentioned, given the experiment design it is still unconvincing that reward amount influences the probability prior, even when including the idea of choice probabilities. In their experiment, the participants are given the predefined priors at the start of each trial. If the participants had not been given the priors at the start of the trial, then the choice probability would factor based on how they explored and experienced different reward probabilities and reward amounts to update trial-by-trial level priors. Perhaps this argument might be made if operating under the assumption that participants do not fully believe that the indicated reward amount and reward probability priors are actually true and being delivered as stated. Furthermore, I would imagine that participants would make similar choices if they were provided reward during free-choice trials.

a. It is suggested that the authors (a) remove the idea that reward amount influences the prior, or (b) clarify the assumptions being made about prior updating and provide a concrete numerical example / formula. Since the arguments made about the dissociation are not actually critical the paper, perhaps (a) would be the preferred choice.

A challenge of comparing reward probability and amount is that they are in different units. Further, probabilities are bounded while amounts can be infinitely large. This can pose a challenge from an interpretation and statistical perspective. From an interpretation perspective, would the results and interpretation similarly hold if the reward amount maximum was extremely large, e.g., $1000.00 per trial? It is conceivable in this situation that the amount may dominate (ignoring expected value). In other words, are the observed trends and message of the paper true because of the limited amount of reward amount being offered to participants? Statistically, the authors normalize the reward probability and reward amount by matching the relative change in amount and probability used in the experiment conditions. While this is a way to make both unitless, the reward amount normalization would be different whenever there is a change in values. It would seem the interpretation is likely limited to the range of reward amounts used within their experiment. Can the authors please comment and, if appropriate, include this as a potential limitation.

Why was it hypothesized “that the PROB prior has a larger influence on movement and choice than the AMNT prior” (p. 14)? Is there a model that could predict these results? (Would the results look different if a different pay-off rule were used, i.e. larger rewards?)

It has also previously been demonstrated that (prior) information about the (relative) target position can be learnt across many hundreds of trails during motor tasks, also involving stochastic rewards (see e.g. Körding and Wolpert, Nature 427 (6971), 244-247 and also, e.g. Seydell et al., 2008, Journal of Neuroscience 28 (17) 4356-4367). In the study here however, the probabilities about the binary target locations were visually communicated at the beginning of trial, but not really experienced by the participants (in the relatively short blocks of 60 to 72 trials). It is hard to imagine that participants did generate an accurate internal estimate of the prior probability presented here and unclear to me in which sense this prior is relevant for target selection (other than inducing a preference for either target location). In other words, in which way are the explicit probabilities stated here meaningful and relevant for motor planning? Would a less visible, but highly probable target induce the same kind of deviation and correction of the movement?

In a previous study, it was shown that hand movements were strongly affected by priming during the movement (Schmidt, 2002, Psychological Science 13 (2), 112-118). In the study here, information about the target was provided visually during the reach. How is this information different from the visual primes used by Schmidt (2002) and how do the observed online corrections observed here compare to previous findings along these lines of research?

How do the results observed here fit into the framework of “cost” of movement (see, e.g. book by Shadmehr & Ahmed, 2020, Vigor MIT Press)?

line 605: a competing theory with parallel plans is a single, flexible plan (see Hudson, T. E., Maloney, L. T., & Landy, M. S. (2007). Movement planning with probabilistic  target information. Journal of Neurophysiology, 98 (5), 3034-3046; Haith, A. M., Huberdeau, D. M., & Krakauer, J. W. (2015). Hedging your bets: intermediate  movements as optimal behavior in the context of an incomplete decision. PLoS Computational  Biology, 11 (3), e1004171; Wong, A. L., & Haith, A. M. (2017). Motor planning flexibly optimizes performance under  uncertainty about task goals. Nature Communications, 8 (1), 1-10; Nashed, J. Y., Diamond, J. S., Gallivan, J. P., Wolpert, D. M., & Flanagan, J. R. (2017).  Grip force when reaching with target uncertainty provides evidence for motor optimization  over averaging. Scientific Reports, 7 (1), 1-8; Alhussein, L., & Smith, M. A. (2021). Motor planning under uncertainty. Elife, 10 , e67019.  

End point distributions can give insight about feedback mechanisms involved (see, e.g. Diedrichsen et al. 2010, Trends Cog. Sciences, 14, 31-39). Are there any systematic changes in end distributions here, e.g. depending on TOC or type of prior?

Are there any trial-by-trial effects, specifically following missed targets?

Were the errors observed constant over the duration of the experiment or were there any learning effects?

Is there any baseline condition of a natural reach (without an initially probabilistic target) to which the results can be compared?

The results for the participants seem quite heterogeneous. Would a correction for the differences in onset time make a difference?

Minor:

How were participants instructed and the visual stimuli explained? Did they receive a written explanation about the meaning of the visually displayed prior information?

Fig. 1: Please state clearly when pre-cue A was presented.

Fig. 2 D: I find the axes labels and titles confusing. What kind of movement time is presented here?

Figs. 2-2 and Fig. 2-3: Please add the number of participants.

Figure 3: This does not seem to influence the interpretation as the effects are likely minimal, but TOC is slightly biased metric since it would require some additional time for participants to overcome inertia of the upper limb when changing direction. Would be useful to mention this potential limitation in the paper.

Line 7. This is stylistic, but the e.g., is slightly awkward. Perhaps, “When deciding while acting, such as sequentially searching and selecting targets during naturalistic behaviours like foraging, movement trajectories can be informative about the unfolding decision process.

line 15: Unsure what, “save to choose freely” means. Perhaps, “.... or freely chosen.”

The abstract would benefit from mentioning expected value.

line 186. ...”were realized...” is a slightly awkward phrasing.

Line 236: how many failed trials were there?

line 247: Did the authors use a dual-pass filter (forward and backward in time) to remove the time delays introduced by the filter. Also, I doubt this would influence the data very much given the butterworth filter, but it does not seem necessary to down sample (or cubic spline which influences the frequency content).

line 250-251: Not sure why the authors used either position or velocity criteria. Can the authors please comment.

Permutation tests can produce variable results unless n is sufficiently high. A suggested minimum is 10,000, preferably 100,000.

lines: 446-454: might be worthwhile to mention that selecting the closer target during free choice reflects an energetic consideration since there is no longer any expected value per target.

## References

[B1] Alhussein L, Smith MA (2021) Motor planning under uncertainty. Elife 10:e67019. 10.7554/eLife.67019 34486520 PMC8421070

[B2] Atiya NAA, Zgonnikov A, Hora DO, Schoemann M, Scherbaum S, Wong-Lin K (2020) Changes-of-mind in the absence of new post- decision evidence. PLoS Comput Biol 16:e1007149. 10.1371/journal.pcbi.1007149 32012147 PMC7018100

[B3] Brenner E, Smeets JBJ (1997) Fast responses of the human hand to changes in target position. J Mot Behav 29:297–310. 10.1080/00222899709600017 12453772

[B4] Burk D, Ingram JN, Franklin DW, Shadlen MN, Wolpert DM (2014) Motor effort alters changes of mind in sensorimotor decision making. PLoS One 9:e92681. 10.1371/journal.pone.0092681 24651615 PMC3961398

[B5] Carroll TJ, Mcnamee D, Ingram JN, Wolpert DM (2019) Rapid visuomotor responses reflect value-based decisions. J Neurosci 39:3906–3920. 10.1523/JNEUROSCI.1934-18.2019 30850511 PMC6520503

[B6] Carsten T, Fievez F, Duque J (2022) Movement characteristics impact decision-making and vice versa. BioRxiv 478832. 10.1101/2022.02.02.478832. PMC9968293 36841847

[B7] Chapman CS, Gallivan JP, Wood DK, Milne JL, Culham JC, Goodale MA (2010) Reaching for the unknown: multiple target encoding and real-time decision-making in a rapid reach task. Cognition 116:168–176. 10.1016/j.cognition.2010.04.008 20471007

[B8] Chapman CS, Gallivan JP, Wong JD, Wispinski NJ, Enns JT (2015) The snooze of lose: rapid reaching reveals that losses are processed more slowly than gains. J Exp Psychol Gen 144:844–863. 10.1037/xge0000085 26097977

[B9] Cisek P, Kalaska JF (2005) Neural correlates of reaching decisions in dorsal premotor cortex: specification of multiple direction choices and final selection of action. Neuron 45:801–814. 10.1016/j.neuron.2005.01.027 15748854

[B10] Cisek P, Kalaska JF (2010) Neural mechanisms for interacting with a world full of action choices. Annu Rev Neurosci 33:269–298. 10.1146/annurev.neuro.051508.135409 20345247

[B11] Cos I, Bélanger N, Cisek P (2011) The influence of predicted arm biomechanics on decision making. J Neurophysiol 105:3022–3033. 10.1152/jn.00975.2010 21451055

[B12] Cos I, Duque J, Cisek P (2014) Rapid prediction of biomechanical costs during action decisions. J Neurophysiol 112:1256–1266. 10.1152/jn.00147.2014 24899673

[B13] Dann B, Michaels JA, Schaffelhofer S, Scherberger H (2016) Uniting functional network topology and oscillations in the fronto-parietal single unit network of behaving primates. Elife 5:1–27. 10.7554/eLife.15719 PMC5019840 27525488

[B14] Dotan D, Pinheiro-Chagas P, Al Roumi F, Dehaene S (2019) Track it to crack it: dissecting processing stages with finger tracking. Trends Cogn Sci 23:1058–1070. 10.1016/j.tics.2019.10.002 31679752

[B15] Freeman JB (2018) Doing psychological science by hand. Curr Dir Psychol Sci 27:315–323. 10.1177/0963721417746793 30581254 PMC6301007

[B16] Friedman J, Brown S, Finkbeiner M (2013) Linking cognitive and reaching trajectories via intermittent movement control. J Math Psychol 57:140–151. 10.1016/j.jmp.2013.06.005

[B17] Gail A (2022) Turning decisions into actions. PLoS Biol 20:e3001927. 10.1371/journal.pbio.3001927 36525393 PMC9757545

[B18] Gallivan JP, Stewart BM, Baugh LA, Wolpert DM, Flanagan JR, Gallivan JP, Stewart BM, Baugh LA, Wolpert DM, Flanagan JR (2017) Rapid automatic motor encoding. Cell Rep 18:1619–1626. 10.1016/j.celrep.2017.01.049 28199835 PMC6103432

[B19] Gallivan JP, Chapman CS, Wolpert DM, Flanagan JR (2018) Decision-making in sensorimotor control. Nat Rev Neurosci 19:519–534. 10.1038/s41583-018-0045-9 30089888 PMC6107066

[B20] Gold JI, Shadlen MN (2007) The neural basis of decision making. Annu Rev Neurosci 30:535–574. 10.1146/annurev.neuro.29.051605.113038 17600525

[B21] Hagura N, Haggard P, Diedrichsen J (2017) Perceptual decisions are biased by the cost to act. Elife 6:e18422. 10.7554/eLife.18422 28219479 PMC5319835

[B22] Haith AM, Huberdeau DM, Krakauer JW (2015) Hedging your bets: intermediate movements as optimal behavior in the context of an incomplete decision. PLoS Comput Biol 11:e1004171. 10.1371/journal.pcbi.1004171 25821964 PMC4379031

[B23] Hudson TE, Maloney LT, Landy MS (2007) Movement planning with probabilistic target information. J Neurophysiol 98:3034–3046. 10.1152/jn.00858.2007 17898140 PMC2638584

[B24] Kahneman D, Tversky A (1979) Prospect theory: an analysis of decision under risk. Econometrica 47:263–292. 10.2307/1914185

[B25] Kim HE, Avraham G, Ivry RB (2021) The psychology of reaching: action selection, movement implementation, and sensorimotor learning. Annu Rev Psychol 72:61–95. 10.1146/annurev-psych-010419-051053 32976728 PMC8514106

[B26] Klaes C, Westendorff S, Chakrabarti S, Gail A (2011) Choosing goals, not rules: deciding among rule-based action plans. Neuron 70:536–548. 10.1016/j.neuron.2011.02.053 21555078

[B27] Körding KP, Wolpert DM (2004) Bayesian integration in sensorimotor learning. Nature 427:244–247. 10.1038/nature02169 14724638

[B28] Leis BC, Rand MK, Van Gemmert AWA, Longstaff MG, Lou JS, Stelmach GE (2005) Movement pre-cues in planning and execution of aiming movements in Parkinson’s disease. Exp Neurol 194:393–409. 10.1016/j.expneurol.2005.02.014 16022867

[B29] Leite FP, Ratcliff R (2011) What cognitive processes drive response biases? A diffusion model analysis. Judgm Decis Mak 6:651–687. 10.1017/S1930297500002680

[B30] Lepora NF, Pezzulo G (2015) Embodied choice: how action influences perceptual decision making. PLoS Comput Biol 11:e1004110. 10.1371/journal.pcbi.1004110 25849349 PMC4388485

[B31] Maris E, Oostenveld R (2007) Nonparametric statistical testing of EEG- and MEG-data. J Neurosci Methods 164:177–190. 10.1016/j.jneumeth.2007.03.024 17517438

[B32] Marti-Marca A, Deco G, Cos I (2020) Visual-reward driven changes of movement during action execution. Sci Rep 10:15527. 10.1038/s41598-020-72220-2 32968102 PMC7511350

[B33] Michalski J, Green AM, Cisek P (2020) Reaching decisions during ongoing movements. J Neurophysiol 123:1090–1102. 10.1152/jn.00613.2019 32049585 PMC7099481

[B34] Mir P, Trender-Gerhard I, Edwards MJ, Schneider SA, Bhatia KP, Jahanshahi M (2011) Motivation and movement: the effect of monetary incentive on performance speed. Exp Brain Res 209:551–559. 10.1007/s00221-011-2583-5 21337028

[B35] Morel P (2018) Gramm: grammar of graphics plotting in Matlab. J Open Source Softw 3:568. 10.21105/joss.00568

[B36] Morel P, Ulbrich P, Gail A (2017) What makes a reach movement effortful? Physical effort discounting supports common minimization principles in decision making and motor control. PLoS Biol 15:e2001323 . 10.1371/journal.pbio.2001323 28586347 PMC5460791

[B37] Mulder MJ, Wagenmakers EJ, Ratcliff R, Boekel W, Forstmann BU (2012) Bias in the brain: a diffusion model analysis of prior probability and potential payoff. J Neurosci 32:2335–2343. 10.1523/JNEUROSCI.4156-11.2012 22396408 PMC6621823

[B38] Nagengast AJ, Braun DA, Wolpert DM (2010) Risk-sensitive optimal feedback control accounts for sensorimotor behavior under uncertainty. PLoS Comput Biol 6:e1000857. 10.1371/journal.pcbi.1000857 20657657 PMC2904762

[B39] Nagengast AJ, Braun DA, Wolpert DM (2011a) Risk sensitivity in a motor task with speed-accuracy trade-off. J Neurophysiol 105:2668–2674. 10.1152/jn.00804.2010 21430284 PMC3118741

[B40] Nagengast AJ, Braun DA, Wolpert DM (2011b) Risk-sensitivity and the mean-variance trade-off: decision making in sensorimotor control. Proc Biol Sci 278:2325–2332. 10.1098/rspb.2010.2518 21208966 PMC3119020

[B41] Nashed JY, Crevecoeur F, Scott SH (2014) Rapid online selection between multiple motor plans. J Neurosci 34:1769–1780. 10.1523/JNEUROSCI.3063-13.2014 24478359 PMC8186509

[B42] Nashed JY, Diamond JS, Gallivan JP, Wolpert DM, Flanagan JR (2017) Grip force when reaching with target uncertainty provides evidence for motor optimization over averaging. Sci Rep 7:11703. 10.1038/s41598-017-10996-6 28916824 PMC5601432

[B43] Pastor-Bernier A, Cisek P (2011) Neural correlates of biased competition in premotor cortex. J Neurosci 31:7083–7088. 10.1523/JNEUROSCI.5681-10.2011 21562270 PMC6703218

[B44] Pezzulo G, Cisek P (2016) Navigating the affordance landscape: feedback control as a process model of behavior and cognition. Trends Cogn Sci 20:414–424. 10.1016/j.tics.2016.03.013 27118642

[B45] Platt ML, Glimcher PW (1999) Neural correlates of decision variables in parietal cortex. Nature 400:233–238. 10.1038/22268 10421364

[B46] Resulaj A, Kiani R, Wolpert DM, Shadlen MN (2009) Changes of mind in decision-making. Nature 461:263–266. 10.1038/nature08275 19693010 PMC2875179

[B47] Scherbaum S, Dshemuchadse M (2020) Psychometrics of the continuous mind: measuring cognitive sub-processes via mouse tracking. Mem Cognit 48:436–454. 10.3758/s13421-019-00981-x 31721062

[B48] Scherbaum S, Dshemuchadse M, Fischer R, Goschke T (2010) How decisions evolve: the temporal dynamics of action selection. Cognition 115:407–416. 10.1016/j.cognition.2010.02.004 20227687

[B49] Seydell A, Mccann BC, Trommershäuser J, Knill DC (2008) Learning stochastic reward distributions in a speeded pointing task. J Neurosci 28:4356–4367. 10.1523/JNEUROSCI.0647-08.2008 18434514 PMC3844797

[B50] Stewart BM, Gallivan JP, Baugh LA, Flanagan JR (2014) Motor, not visual, encoding of potential reach targets. Curr Biol 24:R953–R954. 10.1016/j.cub.2014.08.046 25291634

[B51] Stillman PE, Krajbich I, Ferguson MJ (2020) Using dynamic monitoring of choices to predict and understand risk preferences. Proc Natl Acad Sci U S A 117:31738–31747. 10.1073/pnas.2010056117 33234567 PMC7749332

[B52] Summerside EM, Shadmehr R, Ahmed AA (2018) Vigor of reaching movements: reward discounts the cost of effort. J Neurophysiol 119:2347–2357. 10.1152/jn.00872.2017 29537911 PMC6734091

[B53] Suriya-Arunroj L, Gail A (2015) I plan therefore I choose: free-choice bias due to prior action-probability but not action-value. Front Behav Neurosci 9:315. 10.3389/fnbeh.2015.00315 26635565 PMC4658425

[B54] Suriya-Arunroj L, Gail A (2019) Complementary encoding of priors in monkey frontoparietal network supports a dual process of decision-making. Elife 8:e47581. 10.7554/eLife.47581 31612855 PMC6794075

[B55] Trommershäuser J, Maloney LT, Landy MS (2003a) Rapid, goal-directed movements. J Opt Soc Am A Opt Image Sci Vis 20:1419–1433. 10.1364/josaa.20.001419 12868646

[B56] Trommershäuser J, Maloney LT, Landy MS (2003b) Statistical decision theory and trade-offs in the control of motor response. Spat Vis 16:255–275. 10.1163/156856803322467527 12858951

[B57] Trommershäuser J, Landy MS, Maloney LT (2006) Humans rapidly estimate expected gain in movement planning. Psychol Sci 17:981–988. 10.1111/j.1467-9280.2006.01816.x 17176431

[B58] Trommershäuser J, Maloney LT, Landy MS (2008) Decision making, movement planning and statistical decision theory. Trends Cogn Sci 12:291–297. 10.1016/j.tics.2008.04.010 18614390 PMC2678412

[B59] Tversky A, Kahneman D (1992) Advances in prospect theory: cumulative representation of uncertainty. J Risk Uncertainty 5:297–323. 10.1007/BF00122574

[B60] Ulbrich P, Gail A (2021) The cone method: inferring decision times from single-trial 3D movement trajectories in choice behavior. Behav Res 53:2456–2472. 10.3758/s13428-021-01579-5 33852130 PMC8613081

[B61] Wispinski NJ, Gallivan JP, Chapman CS (2020) Models, movements, and minds: bridging the gap between decision making and action. Ann N Y Acad Sci 1464:30–51. 30312476 10.1111/nyas.13973

[B62] Wong AL, Haith AM (2017) Motor planning flexibly optimizes performance under uncertainty about task goals. Nat Commun 8:14624. 10.1038/ncomms14624 28256513 PMC5337982

