# Supplementary material for: Deciding While Acting—Mid-Movement Decisions Are More Strongly Affected by Action Probability than Reward Amount
Source: eNeuro. 2023 Apr 17;10(4):ENEURO.0240-22.2023. doi: 10.1523/ENEURO.0240-22.2023 (PMC10121079; doi:10.1523/ENEURO.0240-22.2023)
Supplement: Table 1-1 — Trial numbers per session and block for each PROB/AMNT combination. aLeft, Right instruction probability; bleft, right reward amount; cnumber of required hits for left instructed/right instructed/free-choice. †Each row equals one block type. Each block type (PROB condition) is repeated four times. ‡Each column equals one block type. Each block type (AMNT condition) is repeated two times. Download Table 1-1, DOCX file. [file enu-eN-NWR-0240-22-s06.docx]

**Extended Data Table 1-1**

|  |  |  |  | AMNT | | | | |
| --- | --- | --- | --- | --- | --- | --- | --- | --- |
| Per-session total number of required hits for each pre-cue combination |  | PROB |  | 1 : 9^a^ | 2.5 : 7.5 | 5 : 5 | 7.5 : 2.5 | 9 : 1 |
|  |  | 0.25 : 0.75^b^ |  | 8/24/16^c^ | 8/24/16 | 8/24/16 | 8/24/16 | 8/24/16 |
|  |  | 0.5 : 0.5 |  | 16/16/16 | 16/16/16 | 16/16/16 | 16/16/16 | 16/16/16 |
|  |  | 0.75 : 0.25 |  | 24/8/16 | 24/8/16 | 24/8/16 | 24/8/16 | 24/8/16 |
|  |  |  |  |  |  |  |  |  |
|  |  |  |  | AMNT | | | | |
| Per-block number of trials in the PROB-blocked, AMNT trial-wise session^†^ |  | PROB |  | 1 : 9 | 2.5 : 7.5 | 5 : 5 | 7.5 : 2.5 | 9 : 1 |
|  |  | 0.25 : 0.75 |  | 2/6/4 | 2/6/4 | 2/6/4 | 2/6/4 | 2/6/4 |
|  |  | 0.5 : 0.5 |  | 4/4/4 | 4/4/4 | 4/4/4 | 4/4/4 | 4/4/4 |
|  |  | 0.75 : 0.25 |  | 6/2/4 | 6/2/4 | 6/2/4 | 6/2/4 | 6/2/4 |
|  |  |  |  |  |  |  |  |  |
|  |  |  |  | AMNT | | | | |
| Per-block number of trials in the AMNT-blocked, PROB trial-wise session^‡^ |  | PROB |  | 1 : 9 | 2.5 : 7.5 | 5 : 5 | 7.5 : 2.5 | 9 : 1 |
|  |  | 0.25 : 0.75 |  | 4/12/8 | 4/12/8 | 4/12/8 | 4/12/8 | 4/12/8 |
|  |  | 0.5 : 0.5 |  | 8/8/8 | 8/8/8 | 8/8/8 | 8/8/8 | 8/8/8 |
|  |  | 0.75 : 0.25 |  | 12/4/8 | 12/4/8 | 12/4/8 | 12/4/8 | 12/4/8 |
