# Supplementary material for: Deciding While Acting—Mid-Movement Decisions Are More Strongly Affected by Action Probability than Reward Amount
Source: eNeuro. 2023 Apr 17;10(4):ENEURO.0240-22.2023. doi: 10.1523/ENEURO.0240-22.2023 (PMC10121079; doi:10.1523/ENEURO.0240-22.2023)
Supplement: Table 2-1 — ClusP test statistics for Figure 2 B-C. t-Meanoriginal and p-values of the significant clusters displayed in Figure 2, B and C. t-meanoriginal refers to the arithmetic mean over all single-time point t-values that make up the t-sumoriginal of the significant cluster. The p-values that correspond to a t-meanoriginal are computed as the percentile of the t-sumoriginal with respect to the null distribution (see Materials and Methods). Download Table 2-1, DOCX file. [file enu-eN-NWR-0240-22-s07.docx]

**Extended Data Table 2-1**

|  | Trial type | Prior | t-mean_original_ | *p* |
| --- | --- | --- | --- | --- |
| ClusP tests of TCMR curves against zero (Figure 2B) | Instructed | PROB | 6.17 | < .001 |
|  |  | AMNT | 6.23 | < .001 |
|  | Free-choice | PROB | 5.65 | < .001 |
|  |  | AMNT | 5.04 | < .001 |
|  |  |  |  |  |
| ClusP test of *raw* PROB minus AMNT TCMR curves against zero (Figure 2D, top) | Instructed | NA | 4.52 | < .001 |
|  | Free-choice | NA | 4.22 | < .001 |
|  |  |  |  |  |
| ClusP test of *normalized* PROB minus AMNT TCMR curves against zero (Figure 2D, bottom) | Instructed | NA | no sig. clusters | NA |
|  | Free-choice | NA | 2.79 | .048 |
