# Supplementary material for: Deciding While Acting—Mid-Movement Decisions Are More Strongly Affected by Action Probability than Reward Amount
Source: eNeuro. 2023 Apr 17;10(4):ENEURO.0240-22.2023. doi: 10.1523/ENEURO.0240-22.2023 (PMC10121079; doi:10.1523/ENEURO.0240-22.2023)
Supplement: Table 2-2 — t-Test statistics for Figure 2D and Extended Data Figure 2-3. t-Test statistics for the pairwise PROB versus AMNT comparisons of TCMR peak time and peak strength. EDF, Extended data figure. All df value = 19. Download Table 2-2, DOCX file. [file enu-eN-NWR-0240-22-s08.docx]

**Extended Data Table 2-2**

|  | Figure | Trial type | *t* | *p* |
| --- | --- | --- | --- | --- |
| Paired t-test of PROB vs AMNT *peak time* | 2D | Instructed | 1.74 | .10 |
|  |  | Free-choice | 2.47 | .02 |
|  | EDF 2-3A | Instructed | −2.32 | .03 |
|  |  | Free-choice | −1.60 | .13 |
|  | EDF 2-3B | Instructed | 3.04 | .007 |
|  |  | Free-choice | 2.26 | .02 |
|  | EDF 2-3C | Instructed | 1.38 | .18 |
|  |  | Free-choice | 0.47 | .65 |
|  | EDF 2-3D | Instructed | 0.10 | .92 |
|  |  | Free-choice | 0.44 | .67 |
|  |  |  |  |  |
| Paired t-test of PROB vs AMNT *peak strength* | 2D | Instructed | 7.09 | < .001 |
|  |  | Free-choice | 6.28 | < .001 |
|  | EDF 2-3A | Instructed | 2.69 | .01 |
|  |  | Free-choice | 1.88 | .07 |
|  | EDF 2-3B | Instructed | 5.47 | < .001 |
|  |  | Free-choice | 2.72 | .01 |
|  | EDF 2-3C | Instructed | 6.75 | < .001 |
|  |  | Free-choice | 4.13 | < .001 |
|  | EDF 2-3D | Instructed | 5.84 | < .001 |
|  |  | Free-choice | 3.03 | .006 |
