# Supplementary material for: Deciding While Acting—Mid-Movement Decisions Are More Strongly Affected by Action Probability than Reward Amount
Source: eNeuro. 2023 Apr 17;10(4):ENEURO.0240-22.2023. doi: 10.1523/ENEURO.0240-22.2023 (PMC10121079; doi:10.1523/ENEURO.0240-22.2023)
Supplement: Table 3-1 — M2 results (early bias). Results of the GLME M2 fitted onto the early bias data. CI, Confidence interval; LB, lower boundary; UB, upper boundary. Separate models were computed for each trial type. Download Table 3-1, DOCX file. [file enu-eN-NWR-0240-22-s09.docx]

**Extended Data Table 3-1**

| Trial Type | Effect | Estimate | 95% CI | | *p* | Random effect STD |
| --- | --- | --- | --- | --- | --- | --- |
|  |  |  | LB | UB |  |  |
| Instructed | Intercept | 0.01 | 0.005 | 0.02 | < .001 | 0.01 |
|  | PROB | 0.13 | 0.07 | 0.19 | < .001 | 0.14 |
|  | AMNT | 0.07 | 0.03 | 0.11 | < .001 | 0.08 |
|  |  |  |  |  |  |  |
| Free-choice | Intercept | 0.13 | 0.06 | 0.20 | < .001 | 0.15 |
|  | PROB | 0.07 | 0.05 | 0.10 | < .001 | 0.06 |
|  | AMNT | 0.04 | 0.02 | 0.06 | < .001 | 0.05 |
