# Supplementary material for: Deciding While Acting—Mid-Movement Decisions Are More Strongly Affected by Action Probability than Reward Amount
Source: eNeuro. 2023 Apr 17;10(4):ENEURO.0240-22.2023. doi: 10.1523/ENEURO.0240-22.2023 (PMC10121079; doi:10.1523/ENEURO.0240-22.2023)
Supplement: Table 4-1 — M2 results (TOC). Results of the GLME M2 fitted onto the TOC data. CI, Confidence interval; LB, lower boundary; UB, upper boundary. Separate models were computed for each trial type. Download Table 4-1, DOCX file. [file enu-eN-NWR-0240-22-s10.docx]

**Extended Data Table 4-1**

| Trial Type | Effect | Estimate | 95% CI | | *p* | Random effect STD |
| --- | --- | --- | --- | --- | --- | --- |
|  |  |  | LB | UB |  |  |
| Instructed | Intercept | 448.17 | 434.74 | 461.60 | < .001 | 30.39 |
|  | PROB | −57.61 | −70.96 | −44.27 | < .001 | 30.08 |
|  | AMNT | −30.38 | −36.54 | −24.23 | < .001 | 13.72 |
|  |  |  |  |  |  |  |
| Free-choice | Intercept | 424.34 | 402.31 | 446.37 | < .001 | 49.84 |
|  | PROB | −28.95 | −35.88 | −22.01 | < .001 | 13.79 |
|  | AMNT | −13.89 | −17.78 | −9.99 | < .001 | 7.39 |
