# Supplementary material for: Deciding While Acting—Mid-Movement Decisions Are More Strongly Affected by Action Probability than Reward Amount
Source: eNeuro. 2023 Apr 17;10(4):ENEURO.0240-22.2023. doi: 10.1523/ENEURO.0240-22.2023 (PMC10121079; doi:10.1523/ENEURO.0240-22.2023)
Supplement: Table 5-1 — M3 and M4 results. Results of the GLME M3 fitted onto the high-PROB choice proportions and M4 fitted onto the high-AMNT choice proportions. CI, Confidence interval; LB, lower boundary; UB, upper boundary. Download Table 5-1, DOCX file. [file enu-eN-NWR-0240-22-s11.docx]

**Extended Data Table 5-1**

| Model | Effect | Estimate | 95% CI | | *p* | Random effect STD |
| --- | --- | --- | --- | --- | --- | --- |
|  |  |  | LB | UB |  |  |
| M3 | Intercept | 0.54 | 0.40 | 0.67 | < .001 | 0.24 |
|  | AMNT^High-PROB^ | 0.16 | 0.10 | 0.22 | < .001 | 0.13 |
|  |  |  |  |  |  |  |
| M4 | Intercept | 0.68 | 0.40 | 0.97 | < .001 | 0.63 |
|  | AMNT^High^ | 0.06 | −0.04 | 0.17 | .22 | 0.13 |
