# Supplementary material for: Deciding While Acting—Mid-Movement Decisions Are More Strongly Affected by Action Probability than Reward Amount
Source: eNeuro. 2023 Apr 17;10(4):ENEURO.0240-22.2023. doi: 10.1523/ENEURO.0240-22.2023 (PMC10121079; doi:10.1523/ENEURO.0240-22.2023)
Supplement: Table 5-2 — M5 results. Results of the GLME M5 fitted onto the per-PROB/AMNT condition choice probabilities as a function of the normalized movement direction. CI, Confidence interval; LB, lower boundary; UB, upper boundary. Download Table 5-2, DOCX file. [file enu-eN-NWR-0240-22-s12.docx]

**Extended Data Table 5-2**

| Model | Effect | Estimate | 95% CI | | *p* | Random effect STD |
| --- | --- | --- | --- | --- | --- | --- |
|  |  |  | LB | UB |  |  |
| CP PROB/AMNT = 0.75/1 | Intercept | 0.53 | 0.38 | 0.68 | < .001 | 0.19 |
|  | Slope | 2.45 | 1.30 | 3.59 | < .001 | 2.26 |
|  |  |  |  |  |  |  |
| CP PROB/AMNT = 0.75/2.5 | Intercept | 0.67 | 0.44 | 0.89 | < .001 | 0.41 |
|  | Slope | 3.02 | 1.45 | 4.59 | < .001 | 3.21 |
|  |  |  |  |  |  |  |
| CP PROB/AMNT = 0.75/5 | Intercept | 0.97 | 0.64 | 1.31 | < .001 | 0.67 |
|  | Slope | 2.93 | 1.42 | 4.43 | < .001 | 2.93 |
|  |  |  |  |  |  |  |
| CP PROB/AMNT = 0.75/7.5 | Intercept | 1.16 | 0.79 | 1.53 | < .001 | 0.71 |
|  | Slope | 2.63 | 1.25 | 4.01 | < .001 | 2.65 |
|  |  |  |  |  |  |  |
| CP PROB/AMNT = 0.75/9 | Intercept | 1.03 | 0.69 | 1.38 | < .001 | 0.63 |
|  | Slope | 4.03 | 1.88 | 6.17 | < .001 | 4.37 |
|  |  |  |  |  |  |  |
| CP PROB/AMNT = 0. 5/7.5 | Intercept | 0.33 | 0.13 | 0.53 | .001 | 0.33 |
|  | Slope | 3.32 | 1.50 | 5.13 | < .001 | 3.83 |
|  |  |  |  |  |  |  |
| CP PROB/AMNT = 0. 5/9 | Intercept | 0.38 | 0.21 | 0.56 | < .001 | 0.23 |
|  | Slope | 3.31 | 1.69 | 4.93 | < .001 | 3.35 |
|  |  |  |  |  |  |  |
| CP right target PROB/AMNT = 0. 5/5 | Intercept | 0.35 | −0.04 | 0.75 | .08 | 0.75 |
|  | Slope | 2.37 | 0.80 | 3.94 | .003 | 2.75 |
